# Evolution of Endothelin signaling and diversification of adult pigment pattern in *Danio* fishes

**DOI:** 10.1371/journal.pgen.1007538

**Published:** 2018-09-18

**Authors:** Jessica E. Spiewak, Emily J. Bain, Jin Liu, Kellie Kou, Samantha L. Sturiale, Larissa B. Patterson, Parham Diba, Judith S. Eisen, Ingo Braasch, Julia Ganz, David M. Parichy

**Affiliations:** 1 Department of Biology, University of Washington, Seattle, WA, United States of America; 2 Department of Biology and Department of Cell Biology, University of Virginia, Charlottesville, VA, United States of America; 3 Institute of Neuroscience, University of Oregon, Eugene, OR, United States of America; 4 Department of Integrative Biology, Michigan State University, East Lansing, MI, United States of America; University of Pennsylvania School of Medicine, UNITED STATES

## Abstract

Fishes of the genus *Danio* exhibit diverse pigment patterns that serve as useful models for understanding the genes and cell behaviors underlying the evolution of adult form. Among these species, zebrafish *D*. *rerio* exhibit several dark stripes of melanophores with sparse iridophores that alternate with light interstripes of dense iridophores and xanthophores. By contrast, the closely related species *D*. *nigrofasciatus* has an attenuated pattern with fewer melanophores, stripes and interstripes. Here we demonstrate species differences in iridophore development that presage the fully formed patterns. Using genetic and transgenic approaches we identify the secreted peptide Endothelin-3 (Edn3)—a known melanogenic factor of tetrapods—as contributing to reduced iridophore proliferation and fewer stripes and interstripes in *D*. *nigrofasciatus*. We further show the locus encoding this factor is expressed at lower levels in *D*. *nigrofasciatus* owing to *cis*-regulatory differences between species. Finally, we show that functions of two paralogous loci encoding Edn3 have been partitioned between skin and non-skin iridophores. Our findings reveal genetic and cellular mechanisms contributing to pattern differences between these species and suggest a model for evolutionary changes in Edn3 requirements for pigment patterning and its diversification across vertebrates.

## Author summary

Neural crest derived pigment cells generate the spectacular variation in skin pigment patterns among vertebrates. Mammals and birds have just a single skin pigment cell, the melanocyte, whereas ectothermic vertebrates have several pigment cells including melanophores, iridophores and xanthophores, that together organize into a diverse array of patterns. In the teleost zebrafish, *Danio rerio*, an adult pattern of stripes depends on interactions between pigment cell classes and between pigment cells and their tissue environment. The close relative *D*. *nigrofasciatus* has fewer stripes and prior analyses suggested a difference between these species that lies extrinsic to the pigment cells themselves. A candidate for mediating this difference is Endothelin-3 (Edn3), essential for melanocyte development in warm-blooded animals, and required by all three classes of pigment cells in an amphibian. We show that Edn3 specifically promotes iridophore development in *Danio*, and that differences in Edn3 expression contribute to differences in iridophore complements, and striping, between *D*. *rerio* and *D*. *nigrofasciatus*. Our study reveals a novel function for Edn3 and provides new insights into how changes in gene expression yield morphogenetic outcomes to effect diversification of adult form.

## Introduction

Mechanisms underlying species differences in adult form remain poorly understood. Quantitative genetic analyses and association studies have made progress in identifying loci, and even specific nucleotides, that contribute to morphological differences between closely related species and strains. Yet it remains often mysterious how allelic effects are translated into specific cellular outcomes of differentiation and morphogenesis to influence phenotype. Elucidating not only the genes but also the cellular behaviors underlying adult morphology and its diversification remains a persistent challenge at the interface of evolutionary genetics and developmental biology.

To address genes and cellular outcomes in an evolutionary context requires a system amenable to modern methods of developmental genetic analysis and rich in phenotypic variation. Ideally the trait of interest would have behavioral or ecological implications, and its phenotype would be observable at a cellular level during development. In this context, adult pigment patterns of fishes in the genus *Danio* provide a valuable opportunity to interrogate genetic differences and the phenotypic consequences of these differences.

*Danio* fishes exhibit adult pigment patterns that include horizontal stripes, vertical bars, dark spots, light spots, uniform patterns and irregularly mottled patterns [1]. Pattern variation affects shoaling and might plausibly impact mate recognition, mate choice, and susceptibility to predation [2–5]. Phylogenetic relationships among species and subspecies are increasingly well understood, as is their biogeography, and some progress has been made towards elucidating their natural history [1,6–9]. Importantly in a developmental genetic context, one of these species, zebrafish *D*. *rerio*, is a well-established biomedical model organism with the genetic, genomic and cell biological tools that accompany this status. Such tools can be deployed in other danios to understand phenotypic diversification.

**Fig 1:**
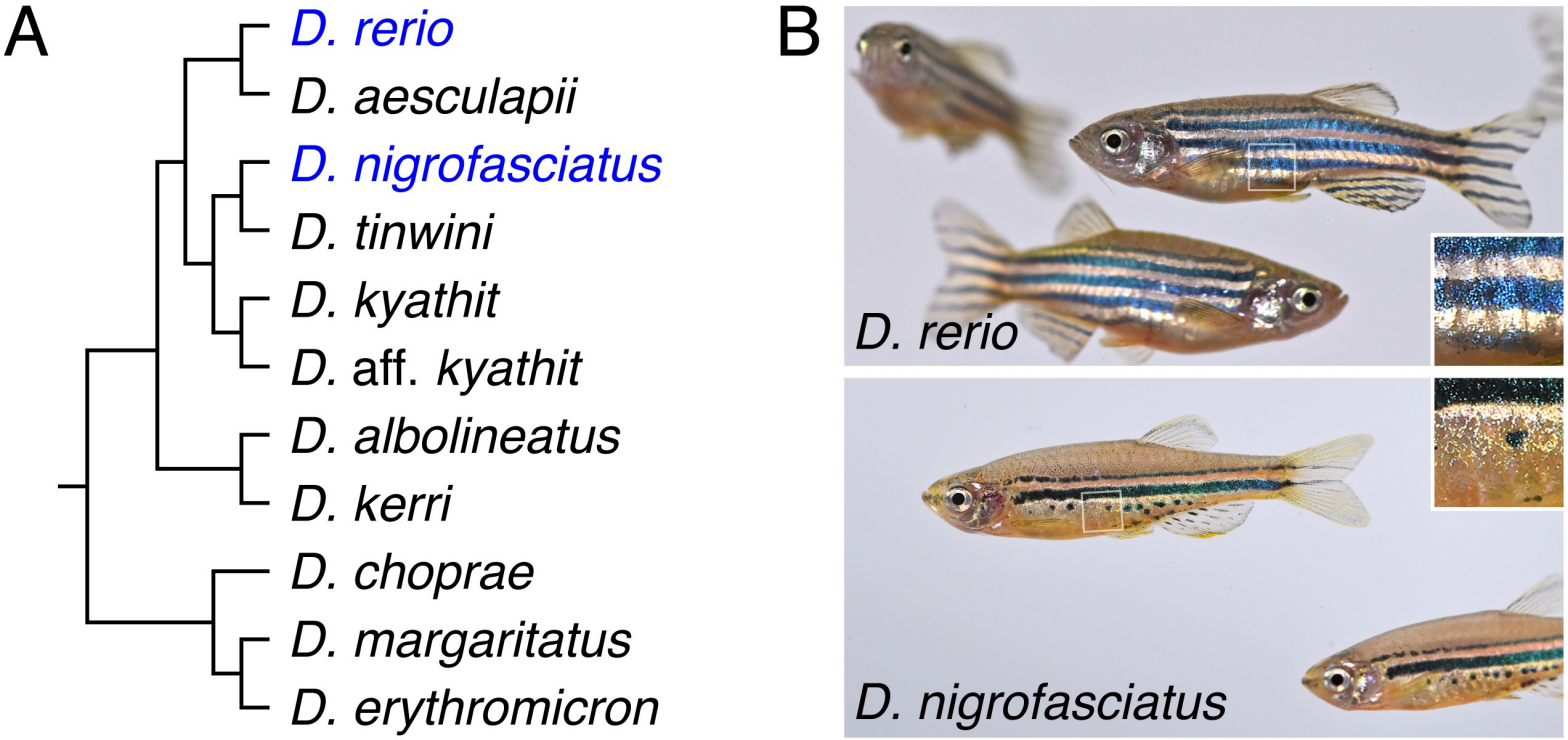
Phylogenetic relationship and pigment patterns of *D*. *rerio* and *D*. *nigrofasciatus*. (A) Phylogenetic relationships of selected *Danio* species [9]. (B) *Danio rerio* exhibit several dark stripes of melanophores with sparse iridophores, and light interstripes with abundant iridophores. *Danio nigrofasciatus* share common pattern elements but have fewer stripes and interstripes overall with spots forming ventrally instead of stripes. A shiny ventrum in both species results principally from iridophores that line the peritoneum, rather than iridophores in the hypodermis of the skin. Insets show iridescence of hypodermal iridophores.

Adult pigment pattern formation in *D*. *rerio* is becoming well described in part because cellular behaviors can be observed directly in both wild-type and genetically manipulated backgrounds. The adult pigment pattern comprises three major classes of pigment cells—black melanophores, iridescent iridophores and yellow–orange xanthophores—all of which are derived directly or indirectly from embryonic neural crest cells [10,11]. The fully formed pattern consists of dark stripes of melanophores and sparse iridophores that alternate with light “interstripes” of xanthophores and dense iridophores (Fig 1, top). During a larva-to-adult transformation, precursors to adult iridophores and melanophores migrate to the skin from locations in the peripheral nervous system [10,12,13]. Once they reach the skin hypodermis, between the epidermis and the underlying myotome, the cells differentiate. Iridophores arrive first and establish a “primary” interstripe near the horizontal myoseptum [14–16]. Differentiating melanophores then form primary stripes dorsal and ventral to the interstripe, with their positions determined in part by interactions with iridophores. Later, xanthophores differentiate within the interstripe and these cells, as well as undifferentiated xanthophores, interact with melanophores to fully consolidate the stripe pattern [11,17–22]. As the fish grows, the pattern is reiterated: loosely arranged iridophores appear within stripes and expand into “secondary” interstripes where they increase in number and establish boundaries for the next forming secondary stripe [13,23]. Stripe development in *D*. *rerio* thus depends on serially repeated interactions among pigment cell classes. It also depends on factors in the tissue environment that are essential to regulating when and where pigment cells of each class appear [11,16].

Analyses of pattern development in other *Danio* are beginning to illuminate how pigment-cell “intrinsic” and “extrinsic” factors have influenced pattern evolution and the genetic bases for such differences [11,21,23–25]. Here, we extend these studies by examining pattern formation in *D*. *nigrofasciatus* (Fig 1, bottom). *D*. *rerio* and *D*. *nigrofasciatus* are closely related and occur within the “*D*. *rerio* species group” [9]. The essential elements of their patterns—stripes and interstripes—and the cell types comprising these patterns are the same. Nevertheless, *D*. *nigrofasciatus* has a smaller complement of adult melanophores than *D*. *rerio* and its stripes are fewer in number, with only residual spots where a secondary ventral stripe would form in *D*. *rerio* [26]. Given the broader distribution of patterns and melanophore complements across *Danio*, the *D*. *nigrofasciatus* pattern of attenuated stripes is likely derived relative to that of *D*. *rerio* and other danios [26,27]. Cell transplantation analyses revealed that species differences in pattern result at least in part from evolutionary alterations residing in the extracellular environment that melanophores experience, rather than factors autonomous to the melanophores themselves [26].

In this study, we show that *D*. *rerio* and *D*. *nigrofasciatus* differ not only in melanophore complements but also iridophore behaviors. We show that iridophore development is curtailed in *D*. *nigrofasciatus*, with a corresponding loss of pattern reiteration. Building on prior inferences [26] and using genetic and transgenic manipulations, we identify the endothelin pathway, and specifically the skin-secreted factor, Endothelin-3 (Edn3), as a candidate for mediating a species difference in iridophore proliferation. We find that *Danio* has two Edn3-encoding loci, arisen from an ancient genome duplication in the ancestor of teleost fishes [28,29], that have diverged in function to promote the development of different iridophore subclasses. One of these, *edn3b*, is required by hypodermal iridophores and has undergone *cis*-regulatory alteration resulting in diminished Edn3 expression in *D*. *nigrofasciatus*. Endothelin signaling is required directly by melanocytes in birds and mammals [30–33] but our findings indicate a specific role for Edn3b in promoting iridophore development, with only indirect effects on melanophores. These results suggest a model for the evolution of Edn3 function across vertebrates and implicate changes at a specific locus, *edn3b*, in altering cellular behavior that determines the numbers of stripes comprising adult pattern.

## Results

### Different iridophore complements of *D*. *nigrofasciatus* and *D*. *rerio*

**Fig 2:**
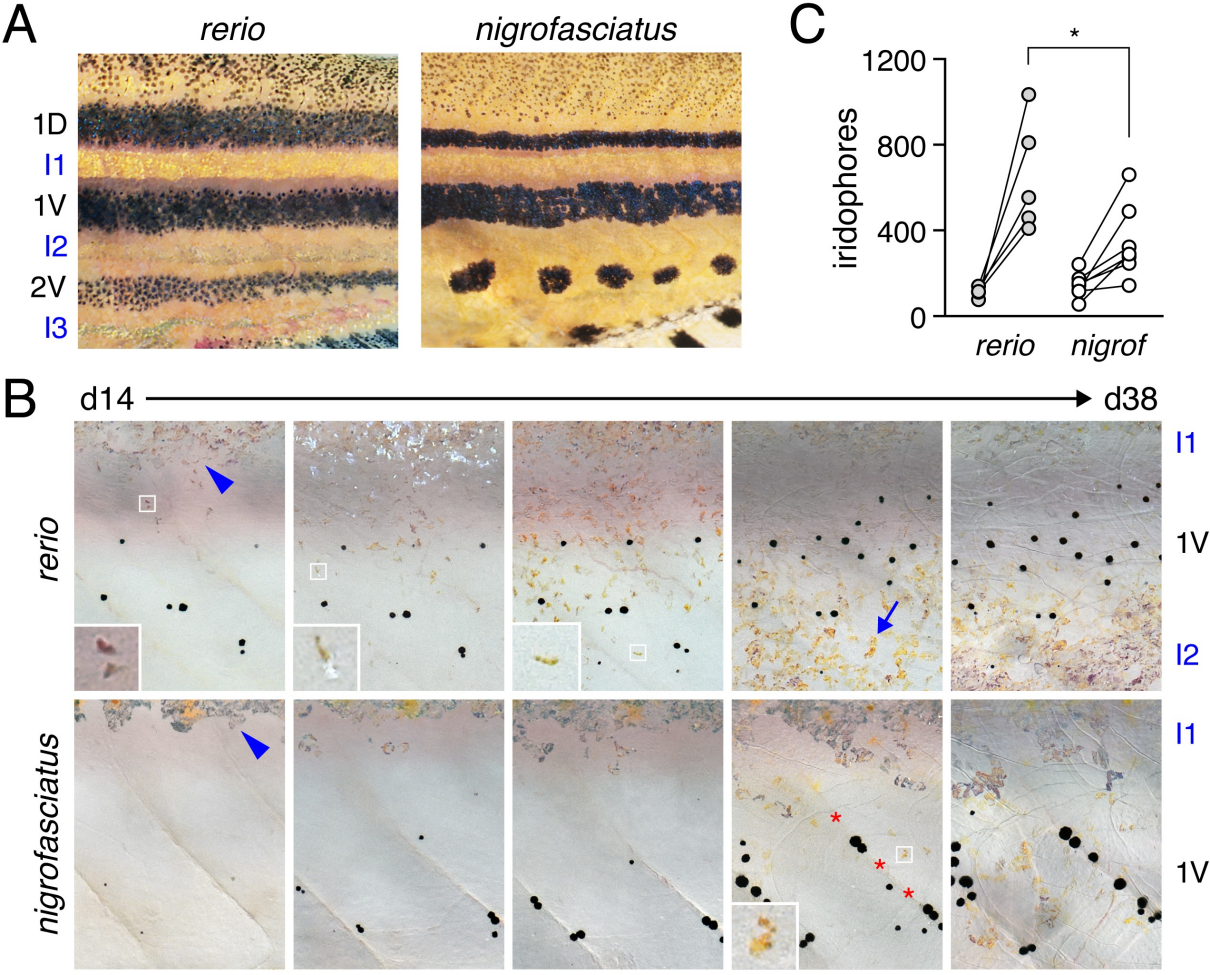
Iridophore development differs between *D*. *rerio* and *D*. *nigrofasciatus*. (A) Young adult patterns of the two species, illustrating fewer melanophores of *D*. *nigrofasciatus* compared to *D*. *rerio*. Stripes and interstripes are marked at the left. 1D, 1V: primary dorsal and ventral stripes. 2V, secondary ventral stripe. I1, I2, I3: Primary, secondary and tertiary interstripes. (B) Iridophores during primary stripe and secondary interstripe formation. Shown are representative individuals imaged repeatedly for *D*. *rerio* (upper) and *D*. *nigrofasciatus* (lower), with iridophores of the primary interstripe indicated by blue arrowheads. Fish were imaged throughout adult pattern formation with stages PB through J [14] illustrated here (corresponding to days ~14 through 38 post fertilization, shown for heuristic purposes only). Insets show iridophores at higher magnification. In *D*. *nigrofasciatus*, iridophores are comparatively few, and do not as extensively populate the region of the secondary ventral stripe or the secondary ventral interstripe (blue arrow in *D*. *rerio*). Melanophores of the primary ventral stripe occur more ventrally than in *D*. *rerio* and tended to be more closely associated with vertical myosepta (marked by red asterisks). Apparent differences in melanophore sizes between species may reflect lower densities and more spread morphologies in *D*. *nigrofasciatus* compared to *D*. *rerio* [26,79]. Sample sizes (*N*): 9 *D*. *rerio*; 6 *D*. *nigrofasciatus*. (C) Clonally related iridophores increased in number in both species between formation of primary interstripe (left; stage PB+) and subsequent pattern reiteration (right; J++). Points connected by lines represent individuals at each developmental stage. Starting numbers were not significantly different (*F*_1,10_ = 0.94, *P* = 0.4), whereas final numbers were significantly fewer in *D*. *nigrofasciatus* than in *D*. *rerio* (repeated measures, species x stage interaction, *F*_1,10_ = 7.47, *P*<0.05; *N* = 5 *D*. *rerio*, *N* = 6 *D*. *nigrofasciatus*).

Iridophores are essential to stripe reiteration of *D*. *rerio* [23] and iridophore-deficient mutants have fewer melanophores [15,16,34]. Given the fewer stripes and melanophores of *D*. *nigrofasciatus* (Fig 2A) [26], we asked whether iridophore development differs in this species from *D*. *rerio*. Fig 2B (upper) illustrates ventral pattern development of *D*. *rerio*. Iridophores were confined initially to the primary interstripe but subsequently occurred as dispersed cells further ventrally [13,16,23]. Additional melanophores developed ventrally to form the ventral primary stripe. Dispersed iridophores were found amongst these melanophores and, subsequently, additional iridophores developed further ventrally as the ventral secondary interstripe. In *D*. *nigrofasciatus*, however, very few dispersed iridophores developed ventral to the primary interstripe (Fig 2B, lower). Melanophores of the prospective ventral primary stripe initially occurred further ventrally than in *D*. *rerio* (also see [26]), similar to mutants of *D*. *rerio* having iridophore defects [16]. Few iridophores were evident either within the prospective ventral primary stripe or further ventrally.

Iridophores arise from progenitors that are established in association with the peripheral nervous system. These cells migrate to the hypodermis where they differentiate [12]. Individual progenitors can generate large hypodermal clones that expand during pattern formation [13]. To assess initial iridophore clone size and subsequent expansion we injected *D*. *rerio* and *D*. *nigrofasciatus* with limiting amounts of *pnp4a*:*palmEGFP* to drive membrane-targeted EGFP in iridophores [21]. At transgene concentrations used, ~1% of injected embryos exhibited a single small patch of EGFP+ iridophores, consistent with labeling of individual progenitors [35,36]. Iridophore morphologies and initial clone sizes were similar between species, but subsequent expansion was significantly greater in *D*. *rerio* than *D*. *nigrofasciatus* (Fig 2C; S1 Fig).

These observations indicate that adult pattern differences between *D*. *rerio* and *D*. *nigrofasciatus* are presaged not only by differences in melanophore development [26] but changes in iridophore behavior as well. This raises the possibility that evolutionary modifications to iridophore morphogenesis or differentiation have contributed to overall pattern differences between species.

### Endothelin pathway mutants identify a candidate gene for the reduced melanophore complement of *D*. *nigrofasciatus*

Shared phenotypes of laboratory variants and other species identify candidate genes that may have contributed to morphological diversification [25,37–44]. *endothelin b1a receptor* (*ednrb1a*) mutant zebrafish resemble *D*. *nigrofasciatus* with deficiencies in iridophores and melanophores compared to wild-type *D*. *rerio*, and a pattern of stripes dorsally with broken stripes or spots ventrally [16,34,45]. Prior genetic analyses failed to identify an obvious role for *ednrb1a* alleles in contributing to these species differences [38]. Ednrb1a is also expressed by pigment cells [34], whereas interspecific cell transplants suggested that pattern differences between *D*. *rerio* and *D*. *nigrofasciatus* likely result from differences in the tissue environment encountered by pigment cells [26]. Given that mutants for Ednrb1a ligand, Endothelin-3 (Edn3), cause pigment cell deficiencies in other vertebrates [46–48], and that Edn3 is likely expressed in the tissue environment of adult pigment cells in *Danio*, we hypothesized that differences in Edn3 expression contribute to the pigment pattern differences between *D*. *rerio* and *D*. *nigrofasciatus*. To first ascertain the phenotype of Edn3 mutants of *D*. *rerio* we induced mutations in each of two Edn3-encoding loci of zebrafish, *edn3a* (chromosome 11) and *edn3b* (chromosome 23) (S2 Fig).

**Fig 3:**
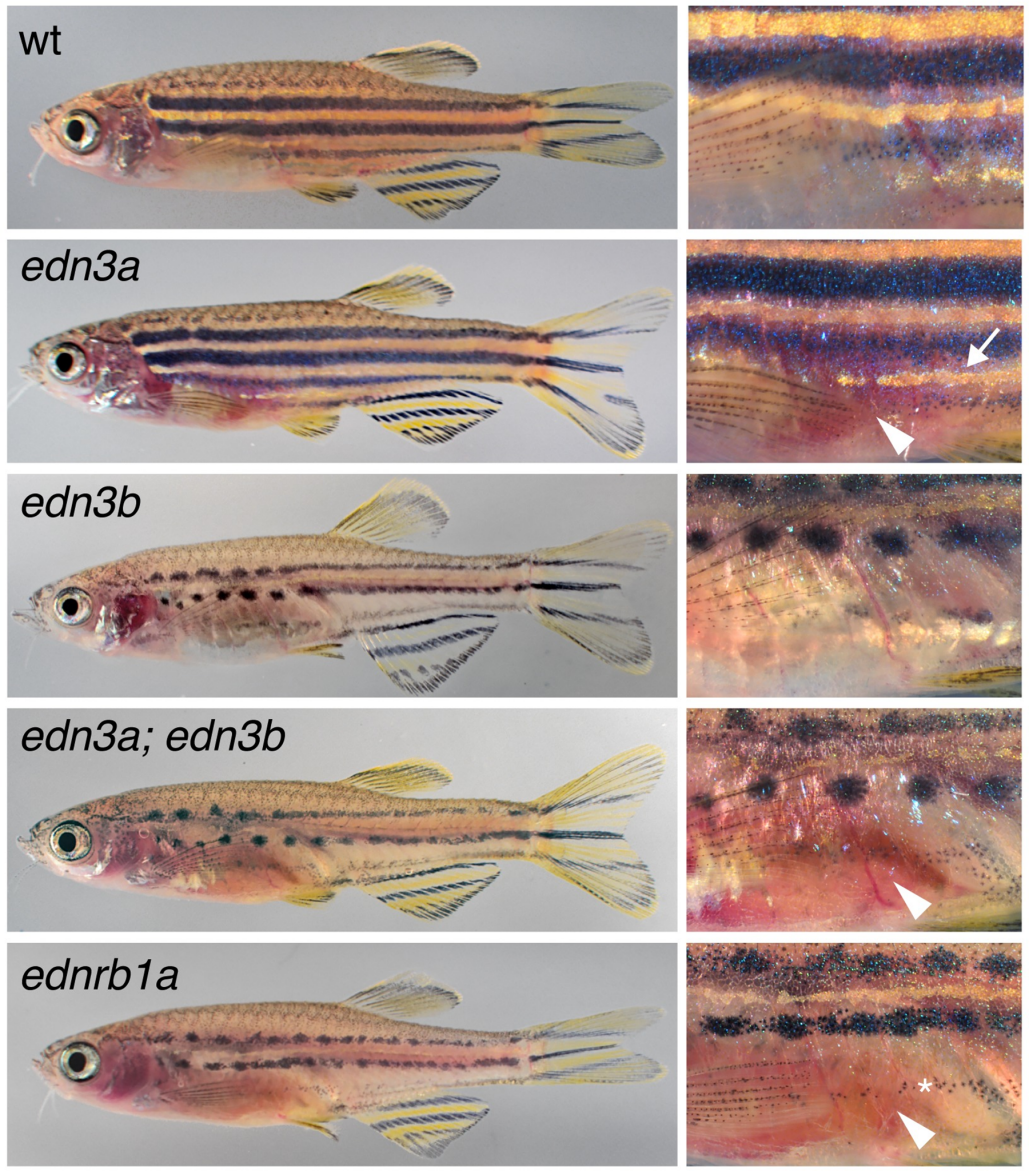
Edn3 and Ednrb1a mutants of *D*. *rerio*. Shown are wild-type (wt) and homozygous mutants for *edn3a* and *edn3b*, double mutant *edn3a; edn3b*, and *ednrb1a*. *edn3a* mutants had normal hypodermal pigment pattern, including iridophore interstripes (arrow, right) but lacked peritoneal iridophores (arrowhead). *edn3b* mutants had hypodermal iridophore and melanophore deficiencies but normal peritoneal iridophores. Fish doubly mutant for these loci exhibited both defects and resembled *ednrb1a* mutants. At the very young adult stages shown, ventral-most melanophores of *ednrb1a* mutants (*) have yet to coalesce into spots.

Fish homozygous mutant for an inactivating allele of *edn3a* exhibited relatively normal stripes and interstripes, but were deficient for iridophores that normally line the peritoneum, resulting in a rosy cast to the ventrum (Fig 3). By contrast, each of three *edn3b* presumptive null alleles exhibited severe deficiencies of hypodermal iridophores and melanophores and patterns of stripes breaking into spots; similar to *D*. *nigrofasciatus*, none had defects in peritoneal iridophores (S3 Fig).

*ednrb1a* mutants are defective for both hypodermal and peritoneal iridophores [34], suggesting that Edn3 signaling may have been partitioned evolutionarily between the two paralogous, ligand-encoding loci. Consistent with this idea, fish doubly mutant for *edn3a* and *edn3b* were deficient for both types of iridophores and resembled mutants for *ednrb1a* (Fig 3). These observations also suggest that Ednrb1a need only interact with Edn3a and Edn3b ligands to fulfill requirements for adult pigmentation, though Ednrb1 receptors of other vertebrate lineages are capable of transducing signals via other endothelins [29].

### Genetic analyses implicate *edn3b* in pattern difference between *D*. *rerio* and *D*. *nigrofasciatus*

The similarity of *edn3b* mutant *D*. *rerio* and *D*. *nigrofasciatus*—with fewer hypodermal melanophores and iridophores than wild-type *D*. *rerio*, but persisting peritoneal iridophores—identified *edn3b* as a particularly good candidate for contributing to the species difference in pigmentation. To assess this possibility further we used an interspecific complementation test [38,42,49]. If a loss-of-function *edn3b* allele contributes to the reduced iridophores and melanophores of *D*. *nigrofasciatus* compared to *D*. *rerio*, we would expect that in hybrids of *D*. *rerio* and *D*. *nigrofasciatus*, substitution of a *D*. *reri*o mutant *edn3b* (*edn3b*^*rerio*–^) allele for a *D*. *rerio* wild-type *edn3b* (*edn3b*^*rerio*+^) allele should expose the “weaker” *D*. *nigrofasciatus* allele, reducing the complement of iridophores and melanophores. Such an effect should be of greater magnitude than substituting a mutant for wild-type allele in *D*. *rerio*, and should be detectable as an allele x genetic background interaction. We therefore generated crosses of *edn3b*/+ *D*. *rerio* x *D*. *nigrofasciatus* as well as *edn3b*/+ x *edn3b*/+ *D*. *rerio*. We grew offspring until juvenile pigment patterns had formed, then genotyped individuals of hybrid (h) or *D*. *rerio* (*r*) backgrounds for the presence of either *edn3b*^*rerio*+^ or *edn3b*^*rerio*–^.

**Fig 4:**
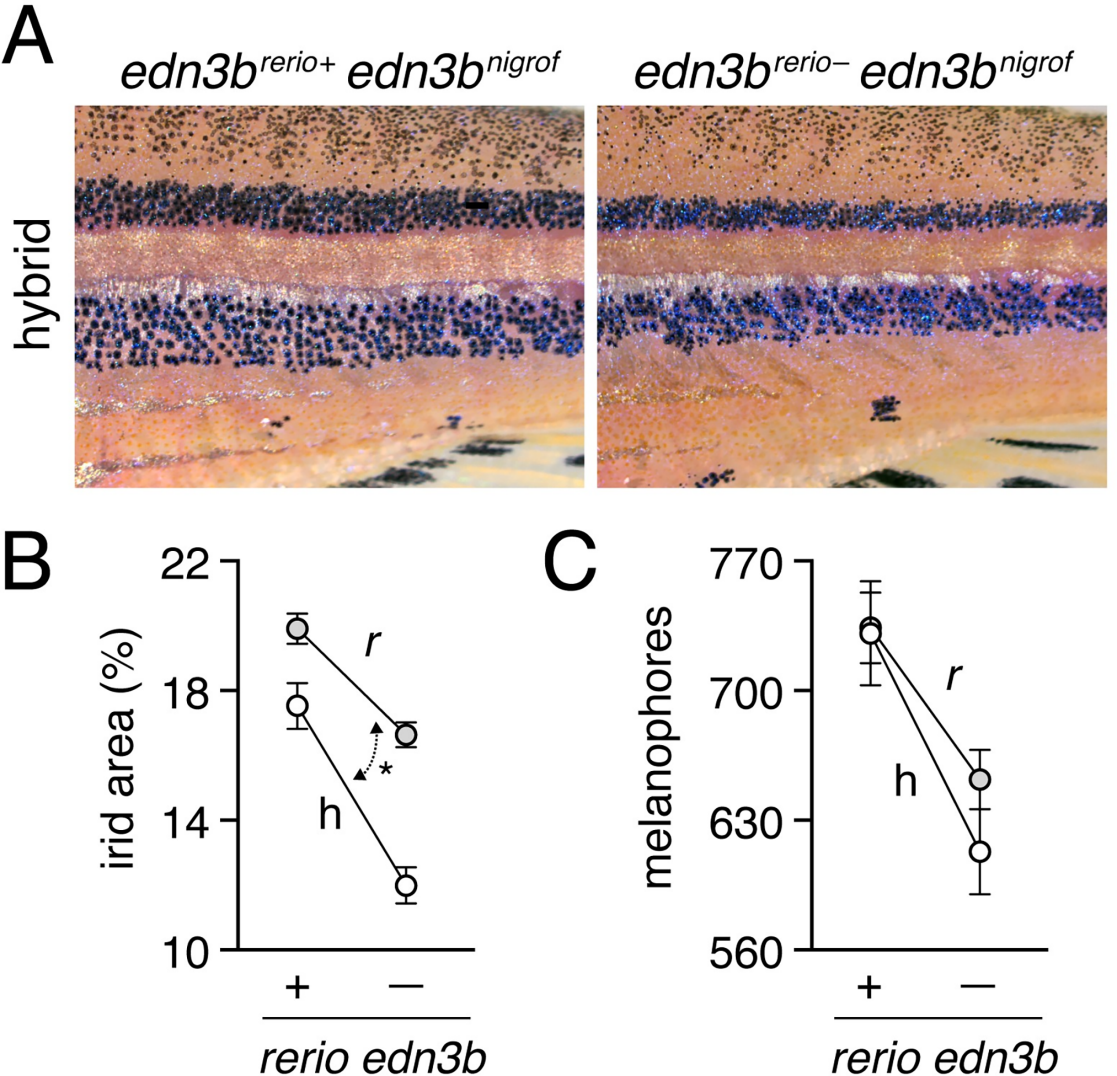
Hypomorphic *edn3b* allele in *D*. *nigrofasciatus* relative to *D*. *rerio*. (A) Interspecific hybrids carrying either *D*. *rerio* wild-type *edn3b* allele (left) or mutant *edn3b* allele (right). Carriers of the mutant allele tended to have narrower stripes and reduced iridophore coverage overall. (B) Hybrids (h) had reduced coverage by dense iridophores of interstripes (total percent of flank) compared to *D*. *rerio* (*r*) overall (*F*_1,59_ = 21.7, P<0.0001). Iridophore coverage was also reduced by substitution of a *D*. *rerio edn3b* mutant allele (–) for the *D*. *rerio* wild-type allele (+; *F*_1,59_ = 101.6, respectively; both *P*<0.0001), but this effect was more pronounced in hybrids, resulting in a significant background x allele interaction (*F*_1,59_ = 6.5, *P*<0.05; double headed arrow, different slopes). (C) Numbers of hypodermal melanophores were affected by background and *D*. *rerio* allele (*F*_1,61_ = 23.5, *F*_1,61_ = 24.7, respectively; both *P*<0.0001), but a background x allele interaction was non-significant (*F*_1,59_ = 1.0, *P* = 0.3). Plots show least squares means±SE after controlling for significant effects of SL (*P*<0.05, *P*<0.0001, respectively). Sample sizes (*N*): 17 *D*. *rerio* (+); 24 *D*. *rerio* (–); 10 hybrids (+); 13 hybrids (–).

Hybrids between *D*. *rerio* and *D*. *nigrofasciatus* have patterns intermediate between the two species [38]. Fig 4A illustrates reduced coverage of iridophores and somewhat narrower stripes in fish carrying *edn3b*^*rerio*–^as compared to siblings carrying *edn3b*^*rerio*+^. Total areas covered by interstripe iridophores were significantly reduced in hybrids compared to *D*. *rerio*, overall, and in both backgrounds by substitution of *edn3b*^*rerio*–^for *edn3b*^*rerio*+^ (Fig 4B). Moreover, hybrids were more severely affected by this substitution than were *D*. *rerio*, resulting in a significant allele x genetic background interaction. Melanophore numbers were also reduced by substitution of *edn3b*^*rerio*–^for *edn3b*^*rerio*+^ but hybrids were not significantly more affected than *D*. *rerio* (Fig 4C). These analyses suggest that the wild-type *D*. *nigrofasciatus edn3b* allele is hypomorphic to the wild-type *D*. *rerio* allele of *edn3b*, and support a model in which evolutionary changes at *edn3b* have affected iridophore coverage between species.

Two other genes, *augmentor-α1a* and *augmentor-α1b*, encoding secreted ligands for Leukocyte tyrosine kinase (Ltk), promote iridophore development in *D*. *rerio* and together have a mutant phenotype resembling *D*. *nigrofasciatus* [50,51]. Iridophore coverage in hybrids carrying *D*. *rerio* mutant alleles of *augmentor-α1a* and *augmentor-α1b* did not differ from siblings carrying *D*. *rerio* wild-type alleles (*F*_1,12_ = 0.01 *P* = 0.9; *F*_1,11_ = 0.3 *P* = 0.6; supplementary Supporting Information), highlighting specificity of the non-complementation phenotype observed for *edn3b*.

### Reduced *edn3b* expression in skin of *D*. *nigrofasciatus* compared to *D*. *rerio* owing to *cis*-regulatory differences

A hypomorphic allele of *edn3b* in *D*. *nigrofasciatus* could result from changes in protein sequence conferring diminished activity, or changes in regulation causing reduced Edn3b abundance. The inferred protein sequence of *D*. *nigrofasciatus* Edn3b did not have obvious lesions (e.g., premature stop codon, deletions or insertions), and the 21 amino acid mature peptide was identical between species.

**Fig 5:**
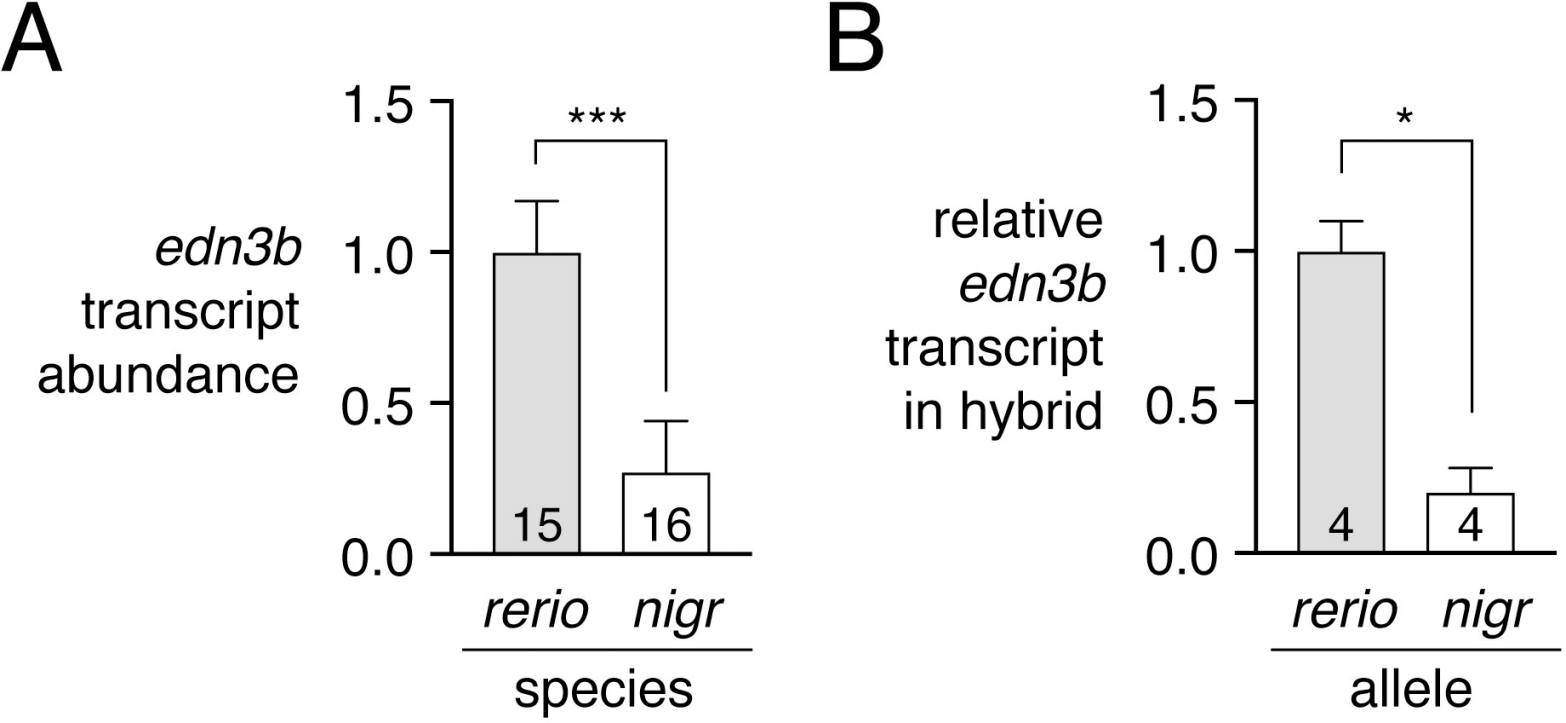
Lower expression of *D*. *nigrofasciatus edn3b* relative to *D*. *rerio edn3b*. (A) *edn3b* was expressed at lower levels in skin of *D*. *nigrofasciatus* than *D*. *rerio* (*F*_1,29_ = 48.6, *P*<0.0001) during adult pattern formation. (B) In hybrid fish, *D*. *nigrofasciatus edn3b* alleles were expressed at lower levels than *D*. *rerio edn3b* alleles (paired *t*_3_ = 4.6, *P*<0.05). Shown are means±SE. Values within bars indicate sample sizes.

We therefore asked whether *D*. *nigrofasciatus edn3b* might be expressed differently than the *D*. *rerio* allele. Presumably owing to low overall levels of expression, *edn3b* transcripts were not detectable by *in situ* hybridization, and transgenic reporters utilizing presumptive regulatory regions amplified by PCR (~5 kb) or contained within bacterial artificial chromosomes (~190 kb containing ~105 kb upstream to the transcriptional start) failed to yield detectable fluorescence, precluding the assessment of spatial variation in gene expression. Nevertheless, quantitative RT-PCR on isolated skins of post-embryonic larvae indicated *edn3b* expression in *D*. *nigrofasciatus* at levels approximately one-quarter that of *D*. *rerio* (Fig 5A). Expression of *edn3b* was similarly reduced in the sister species of *D*. *nigrofasciatus*, *D*. *tinwini*, which has fewer melanophores and iridophores than *D*. *rerio*, and a spotted rather than striped pattern (S4 Fig) [1,9].

This difference in *edn3b* expression raised the possibility that *cis*-regulatory factors (e.g, transcription factor binding sites, chromatin accessibility at *edn3b*) have been altered between *D*. *rerio* and *D*. *nigrofasciatus*. To test this idea, we compared expression of *D*. *rerio* and *D*. *nigrofasciatus edn3b* alleles in the common *trans*-regulatory background of *D*. *rerio* x *D*. *nigrofasciatus* hybrids. Allele-specific quantitative RT-PCR revealed approximately one-quarter the abundance of *D*. *nigrofasciatus edn3b* transcript compared to *D*. *rerio edn3b* transcript (Fig 5B). These observations suggest that species differences in *edn3b* result at least in part from *cis*-regulatory variation that drives lower levels of *edn3b* transcription in *D*. *nigrofasciatus* compared to *D*. *rerio*.

### Edn3b promotes increased iridophore coverage and secondarily affects melanophore pattern in *D*. *nigrofasciatus*

If lower expression of *edn3b* contributes to the difference in pigment pattern between *D*. *nigrofasciatus* and *D*. *rerio*, then expressing *edn3b* at higher levels in *D*. *nigrofasciatus* should generate a pattern converging on that of *D*. *rerio*. To test this prediction, we constructed stable transgenic lines in both species to express *D*. *rerio* Edn3b linked by viral 2A sequence to nuclear-localizing Venus, driven by the ubiquitously expressed heat-shock inducible promoter of *D*. *rerio hsp70l* [16,23]. We then reared *D*. *rerio* and *D*. *nigrofasciatus* transgenic for *hsp70l*:*edn3b-2a-nlsVenus*, and their non-transgenic siblings, under conditions of repeated heat shock during adult pigment pattern formation.

**Fig 6:**
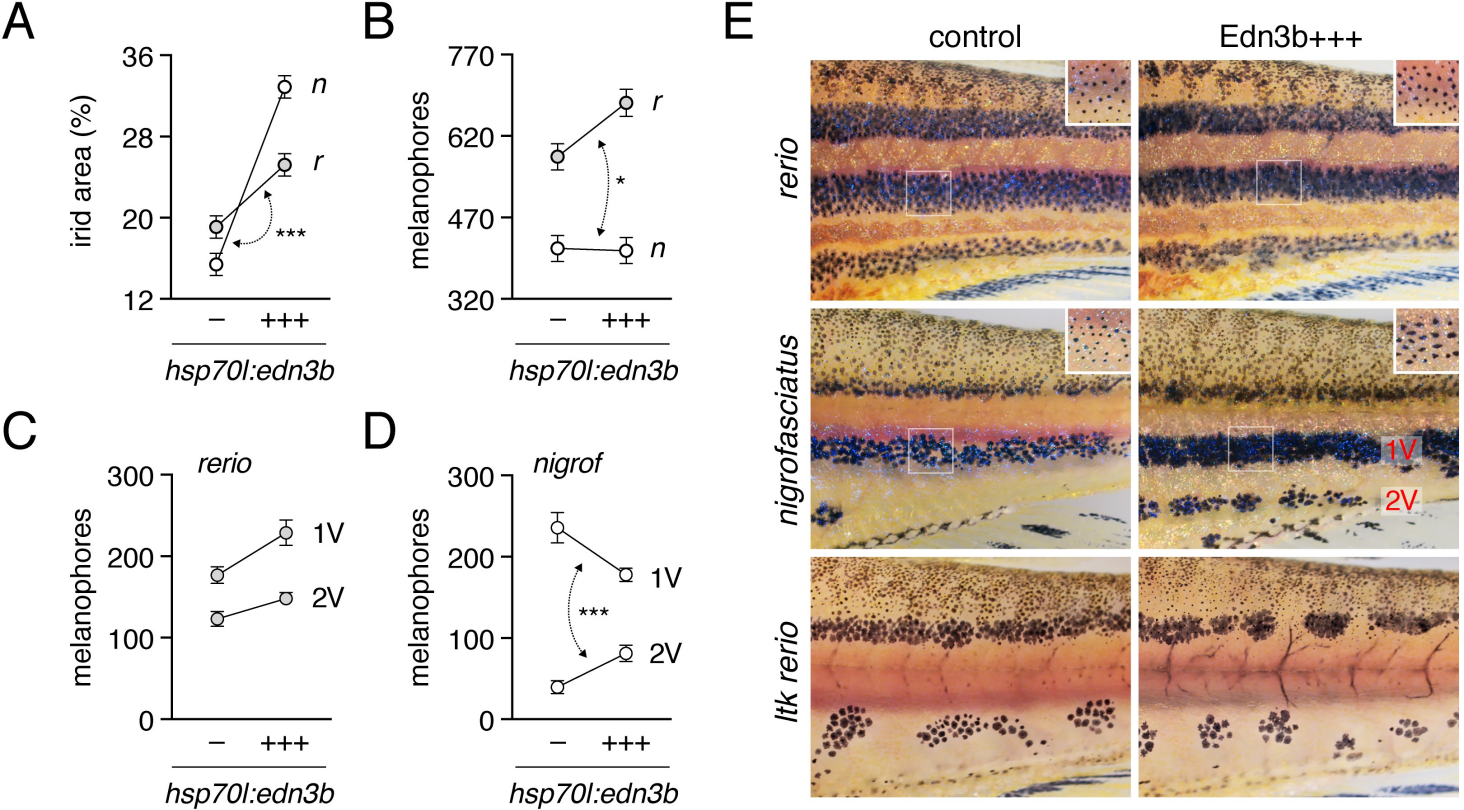
Edn3b increases iridophore coverage in both species and affects melanophore distribution indirectly in *D*. *nigrofasciatus*. (A) In both *D*. *rerio* (*r*) and *D*. *nigrofasciatus* (*n*), relative areas of the flank covered by interstripe (dense) iridophores was increased in response to Edn3b overexpression (+++) as compared to non-transgenic (–) sibling controls treated identically. The response to Edn3b overexpression was more pronounced in *D*. *nigrofasciatus* than in *D*. *rerio* (species x transgene interaction, *F*_1,55_ = 26.49, *P*<0.0001; double headed arrow, different slopes) (B) Edn3b overexpression increased total numbers of hypodermal melanophores in *D*. *rerio* but not *D*. *nigrofasciatus* (species x transgene interaction, *F*_1,55_ = 4.7, *P*<0.05). (C,D) Distributions of melanophores in ventral primary (1V) and ventral secondary (2V) stripes of *D*. *rerio* (C) and *D*. *nigrofasciatus*. In *D*. *nigrofasciatus*, Edn3b overexpression did not increase the total numbers of melanophores in these stripes (*F*_1,28_ = 0.4, *P* = 0.5) but did result in a reallocation of melanophores from 1V to 2V (paired comparison within individuals, stripe position x transgene interaction (*F*_1,26_ = 71.0, *P*<0.0001). All plots show means±SE. (E) Top and middle panels, Phenotypes of each species with and without Edn3b overexpression. In addition to changes in iridophore coverage and melanophore distribution, melanophores of *D*. *nigrofasciatus* overexpressing Edn3b tended to have more dispersed melanin than melanophores of *D*. *nigrofasciatus* controls. Insets show details of 1V stripes following epinephrine treatment, which contracts melanin granules to cell centers and more clearly reveals individual melanophores. All melanophore counts were performed on images of epinephrine-treated fish. Lower panels, Iridophore-free *ltk* mutant *D*. *rerio* in which Edn3b overexpression did not affect the numbers of melanophores (*F*_1,27_ = 1.5, *P* = 0.2) or their allocation between regions (paired comparison within individuals, stripe position x transgene interaction, *F*_1,27_ = 1.5, *P* = 0.2). Positions of stripe breaks and spots were variable among *ltk* mutants and were not consistently altered depending on Edn3b overexpression. Sample sizes (*N*): 15 *D*. *rerio* (–); 15 *D*. *rerio* (+++); 15 *D*. *nigrofasciatus* (–); 15 *D*. *nigrofasciatus* (+++); 15 *ltk* mutant *D*. *rerio* (–); 15 *ltk* mutant *D*. *rerio* (+++).

Heat-shock enhanced expression of Edn3b increased iridophore coverage in *D*. *nigrofasciatus* as compared to *D*. *rerio* or non-transgenic siblings of either species (Fig 6A and 6E). Excess Edn3b failed to increase total numbers of melanophores in *D*. *nigrofasciatus* (Fig 6B). Nevertheless melanophores were differentially distributed in these fish, as *D*. *nigrofasciatus* overexpressing Edn3b had about twice as many cells localizing in a secondary ventral stripe (2V), and a correspondingly reduced number of cells in the primary ventral stripe (1V), as compared to control siblings (Fig 6D). In *D*. *rerio*, total melanophore numbers were increased by Edn3b overexpression though melanophore distributions were not differentially affected between its normally complete stripes (Fig 6B, 6C and 6E).

The rearrangement of a constant number of melanophores in *hsp70l*:*edn3b-2a-nlsVenus D*. *nigrofasciatus*, and a requirement for interactions between iridophores and melanophores during normal stripe formation in *D*. *rerio* [15,16,23], raised the possibility that Edn3b effects on melanophores might be largely indirect, and mediated through iridophores. If so, we predicted that in a background entirely lacking iridophores, *hsp70l*:Edn3b should fail to affect melanophore numbers or distribution. We therefore generated fish transgenic for *hsp70l*:*edn3b-2a-nlsVenus* and homozygous for a mutant allele of *leucocyte tyrosine kinase* (*ltk*), which acts autonomously to promote iridophore development [15,50]. Consistent with iridophore-dependent Edn3b effects, neither melanophore numbers nor melanophore distributions differed between transgenic and non-transgenic siblings (Fig 6E, bottom panels).

These findings support a model in which lower expression of *edn3b* in *D*. *nigrofasciatus* results in diminished coverage by iridophores and a resulting failure of melanophores to more fully populate the secondary ventral stripe, as compared to *D*. *rerio*.

### Iridophore proliferation is curtailed in *D*. *nigrofasciatus* and *edn3b* mutant *D*. *rerio*

Finally, we sought to better understand the cellular bases for Edn3 effects on iridophore populations in *D*. *rerio* and *D*. *nigrofasciatus*. Given roles for Edn3 in promoting the proliferation of avian and mammalian neural crest cells and melanocytes [52–54], we hypothesized that *Danio* Edn3b normally promotes iridophore proliferation and we predicted that such proliferation would be curtailed in both *edn3b* mutant *D*. *rerio* and in *D*. *nigrofasciatus*.

**Fig 7:**
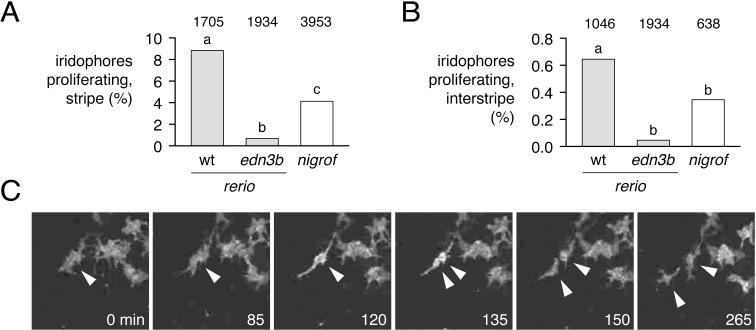
Reduced iridophore proliferation in *edn3b* mutant *D*. *rerio* and *D*. *nigrofasciatus*. (A) Among loosely organized iridophores of prospective stripe regions, the percent of individual cells dividing during time-lapse imaging (15 h total duration) was greatest in *edn3*/+ (wt) *D*. *rerio* and markedly reduced in sibling *edn3b* mutant *D*. *rerio* as well as *D*. *nigrofasciatus* (logistic regression: genotype, χ^2^ = 77.5, d.f. = 2, *P*<0.0001; SL, χ^2^ = 77.6, d.f. = 1, *P*<0.0001). (B) These same trends were evident for densely arranged iridophores of interstripes, though proliferation overall was reduced in comparison to stripe iridophores (genotype, χ^2^ = 13.7, d.f. = 1, *P*<0.005; SL, χ^2^ = 31.9 d.f. = 1 *P*<0.0001). Values above bars indicate total numbers of iridophores examined. Preliminary analysis did not reveal significant variation among individual larvae, the cells of which were pooled for final analyses (larval numbers: 8 *edn3*/+ *D*. *rerio*; 8 *edn3b* mutant *D*. *rerio*; 9 *D*. *nigrofasciatus*). Different letters above bars indicate genotypes that differed significantly from one another in pairwise comparisons of odds ratios (all *P*<0.005). (C) Stills from time-lapse video illustrating a single iridophore (arrowhead) within a prospective stripe region that partially rounds up by 120 min and then divides.

To test these predictions, we examined iridophore behaviors by time-lapse imaging of larvae in which iridophores had been labeled mosaically with a *pnp4a*:*palm-mCherry* transgene. We detected iridophore proliferation in stripe regions, where these cells are relatively few and dispersed, and also within interstripes, where iridophores are densely packed (Fig 7). Proliferation of stripe-region iridophores was ~10-fold greater than that of interstripe iridophores. But within each region, iridophores of wild-type (*edn3b*/+) *D*. *rerio* were more likely to divide than were iridophores of *edn3b* mutants. Iridophores of *D*. *nigrofasciatus* had a proliferative phenotype intermediate to those of wild-type and *edn3b* mutant *D*. *rerio*. We did not observe gross differences in the survival or migration of iridophores across genetic backgrounds. These findings are consistent with Edn3b-dependent differences in iridophore proliferation affecting pattern formation both within *D*. *rerio*, and between *D*. *rerio* and *D*. *nigrofasciatus*.

## Discussion

Towards a fuller understanding of pigment pattern diversification, we have analyzed cellular and genetic bases for differences in adult pattern between *D*. *rerio* and *D*. *nigrofasciatus*. Our study uncovers evolutionary changes in iridophore behavior between these species, identifies endothelin signaling as a candidate pathway contributing to these changes, and provides new insights into the evolution of endothelin genes and functions.

### Evolution of iridophore behaviors and impact on pattern reiteration

An important finding of our analyses is that evolutionary alterations in iridophore behavior can drive species differences in overall pattern. *D*. *rerio* and *D*. *nigrofasciatus* have relatively similar complements of iridophores during early stages of adult pattern formation, but the two species subsequently diverge from one another. In *D*. *rerio*, iridophore clone sizes expanded markedly as the fish grew and secondary and tertiary interstripes were added, whereas this expansion—and pattern element reiteration—were curtailed in *D*. *nigrofasciatus*. The difference in clonal expansion reflected, at least in part, differences in iridophore proliferation as revealed by time-lapse imaging.

Prior efforts documented the essential function of iridophores in promoting melanophore stripe reiteration [16,23]. Here, we showed that enhancing the iridophore complement of *D*. *nigrofasciatus* by Edn3b overexpression was sufficient to reallocate melanophores from a well-formed primary ventral stripe into an otherwise vestigial secondary ventral stripe, resulting in a pattern more like that of *D*. *rerio*. This effect was probably mediated by interactions between iridophores and melanophores, as melanophores did not respond to the same transgene in the *ltk* mutant of *D*. *rerio*, which lacks iridophores. An indirect role for endothelin signaling in promoting melanophore stripe development has likewise been inferred from cell transplantation between wild-type and *ednrb1a* mutant *D*. *rerio* [15], despite expression of *ednrb1a* by newly differentiating melanophores [34] and a responsiveness of *D*. *rerio* melanoma cells to Edn3b in the absence of iridophores [55].

Our observations suggest that an early cessation of iridophore clonal expansion in *D*. *nigrofasciatus* has led to an earlier offset of interactions between iridophores and melanophores, and an attenuation of the stripe pattern in *D*. *nigrofasciatus*. In heterochronic terms, the *D*. *nigrofasciatus* pattern could thus be described as paedomorphic relative to an inferred ancestral state, and arising by progenesis, relative to overall somatic development [56]. That a temporal change in the availability of interactions with iridophores has cascading effects on pattern is reminiscent of observations for xanthophores: precocious widespread xanthophore development, and resulting xanthophore–melanophore interactions, are associated with fewer stripes and more uniform pattern in *D*. *rerio* and *D*. *albolineatus* [23]. These outcomes highlight the diversity of patterns that can arise from a common set of cellular interactions in response to evolutionary modifications to the temporal or spatial pattern of pigment cell appearance.

### A role for endothelin signaling in *Danio* pattern evolution

The numerous pigment mutants of *D*. *rerio* might be expected to include genes that have contributed to evolutionary diversification within *Danio*, particularly when patterns of mutants and species resemble one another. We found that *edn3b* mutants of *D*. *rerio* have fewer iridophores and pattern elements than wild-type *D*. *rerio*, similar to the naturally occurring pattern of *D*. *nigrofasciatus*. This similarity of final phenotype was presaged by similarity of developmental phenotype, as both *edn3b* mutant *D*. *rerio* and *D*. *nigrofasciatus* had reduced iridophore proliferation relative to wild-type *D*. *rerio*.

Our study provides several lines of evidence to support a model in which alterations affecting Edn3b have contributed to the species difference in pigmentation. First, hybrids of *D*. *rerio* and *D*. *nigrofasciatus* carrying a loss-of-function mutant *D*. *rerio* allele of *edn3b* had a more severe iridophore deficiency than heterozygous *D*. *rerio* carrying the same mutant allele, suggesting that the *D*. *nigrofasciatus* wild-type allele is weaker than the *D*. *rerio* wild-type allele. Second, *edn3b* overexpression was sufficient to increase iridophore coverage, and (indirectly) alter melanophore distributions in *D*. *nigrofasciatus* to a state more similar to that of *D*. *rerio*. Third, we found reduced expression of *edn3b* in skin of *D*. *nigrofasciatus* compared to *D*. *rerio* during adult pigment pattern formation. Fourth, species differences in expression of *edn3b* alleles were re-capitulated even in a shared hybrid genetic background, pointing to evolutionary change in *cis*-regulation of this locus. Both *D*. *nigrofasciatus* and *D*. *tinwini* exhibited lower levels of *edn3b* expression compared to *D*. *rerio* so regulatory alteration(s) likely occurred prior to divergence of *D*. *nigrofasciatus* and *D*. *tinwini*, or within the lineage leading to *D*. *rerio* itself. *cis*-regulatory evolution affecting abundance of a secreted ligand that acts on pigment cells to affect pattern is similar to xanthogenic factor Csf1a of *Danio* [23], melanogenic Kit ligand of stickleback [57], and some aspects of anti-melanogenic Agouti in deer mice [58].

Our findings support a role for *edn3b* in *Danio* pattern evolution yet they also point to roles for additional factors. For example, overexpression of Edn3b in *D*. *nigrofasciatus* increased the coverage of iridophores and allowed for some rearrangements of melanophores, but failed to entirely recapitulate the pattern of *D*. *rerio*. Indeed, melanophore numbers were unchanged in transgenic *D*. *nigrofasciatus*, in contrast to the larger overall numbers of melanophore in wild-type *D*. *rerio* and the still larger number of melanophores induced indirectly by Edn3b overexpression in *D*. *rerio* (Fig 6B). Thus, pigment pattern differences between these species are clearly polygenic, and it seems likely that additional loci, of the endothelin pathway or other pathways, will be identified as contributing to attenuated stripes and interstripes of *D*. *nigrofasciatus* compared to *D*. *rerio*.

The endothelin pathway has been implicated in naturally arising strain differences previously. Besides the spontaneous mutant alleles of mouse Edn3 and Ednrb that allowed the pathway to be first characterized molecularly [47,59], endothelin pathway genes or differences in their expression have been associated with tabby coloration in domestic and wild cats [60], melanocyte deficiency in ducks [61], white and hyper-melanistic variants of chicken [62–64] and the white mutant axolotl [48]. It is tempting to speculate that mild alleles of endothelin pathway genes or alterations that affect their expression have relatively few pleiotropic effects, particularly in *Danio*, in which functions of Edn3 paralogues have become subdivided between distinct classes of iridophores. Pigmentary phenotypes associated with this pathway may be particularly accessible targets for natural or artificial selection.

### Evolution of endothelin genes and functions

Finally, our investigation of Edn3b bears on our understanding of how the endothelin pathway and its functions have evolved. Endothelins were discovered for their roles in vasoconstriction and have since been identified to have a variety of functions [29]. In the context of pigmentation, endothelins and their receptors have been most extensively studied in mammals and birds, in which they regulate proliferation, migration, differentiation and survival at various points within the neural crest–melanocyte lineage [30,31,33]. In teleosts, our results in *Danio* suggest that Edn3 acts primarily to promote iridophore development, with only indirect effects on melanophores. By contrast, the salamander *Ambystoma mexicanum* requires *edn3* for the development of melanophores, xanthophores and iridophores [48,65,66] and such effects are not plausibly mediated through iridophores, which develop long after the requirement by melanophores and xanthophores is first manifested.

In teleosts, an additional round of whole genome duplication has resulted in extra genes as compared to non-teleost vertebrates [67–69]. Though many duplicated genes have been lost, those having roles in pigmentation, including genes of the endothelin pathway have been differentially retained [28,29,70–72], presumably owing to the partitioning of ancestral functions and the acquisition of new functions. Our finding that *edn3a* and *edn3b* are required by complementary subsets of iridophores is consistent with subfunctionalization of an ancestral locus required by all iridophores.

Given requirements for Edn3 in other species—and our findings in *Danio* that *edn3a* and *edn3b* are required by iridophores, *edn3b* is required only indirectly by melanophores, and neither locus is required by xanthophores—we can propose a model for functional evolution in which: (i) an ancestral vertebrate Edn3 locus promoted the development of all three classes of pigment cells in ectotherms (a situation currently represented by *A*. *mexicanum*); (ii) loss of iridophores and xanthophores in mammals and birds obviated an Edn3 role in these cell lineages; (iii) Edn3 functional requirements became limited to iridophores in the lineage leading to teleost fishes and then were further partitioned between iridophore populations, at least in *Danio*. Further testing of this scenario will benefit from analyses of additional anamniotes, including gar, which diverged from the teleost lineage prior to the teleost genome duplication [69,73] and might be expected to have an Edn3 requirement similar to that of *A*. *mexicanum*.

## Materials and methods

### Ethics statement

All animal research was conducted according to federal, state and institutional guidelines and in accordance protocols approved by Institutional Animal Care and Use Committees at University of Washington, University of Virginia and University of Oregon. Anesthesia and euthanasia used MS-222

### Fish stocks and rearing conditions

Fish were reared under standard conditions (14L:10D at ~28°C) and staging followed [14]. *Danio rerio* were inbred wild-type WT(ABb), a derivative of AB*. CRISPR/Cas9 mutants were induced in WT(ABb) (*edn3b*^*vp*.*r30c1*^) or ABC x TU (*edn3a*^*b1282*^, *edn3b*^*b1283*^). *Danio nigrofasciatus* was field-collected in Myanmar in 1998 [38] and maintained in the laboratory since that time. *Danio tinwini* was obtained from the pet trade in 2014. Transgenic lines *hsp70l*:*edn3b-2a-nlsVenus*^*vp*.*rt30*^ and *hsp70l*:*edn3b-2a-nlsVenus*^*vp*.*nt2*^ were generated in WT(ABb) and *D*. *nigrofasciatus* backgrounds, respectively. *augmentor-α1a*/+ and *augmentor-α1b*/+ *D*. *rerio* [51] were generously provided by E. Mo and S. Nicoli (Yale School of Medicine). *ltkj*^*9s1*^ (*primrose*) is a spontaneous allele of *ltk* identified by S. Johnson, into which *hsp70l*:*edn3b-2a-nlsVenus*^*vp*.*rt30*^ was crossed.

Fish were fed marine rotifers, brine-shrimp and flake food. Fish were allowed to spawn naturally or gametes were stripped manually for *in vitro* fertilization. Interspecific hybrids were generated by *in vitro* fertilization in both directions using *D*. *rerio* heterozygous for wild-type and *edn3b*^*vp*.*r30c1*^ allele; progeny were reared through formation of juveniles patterns and then genotyped using primers to amplify *D*. *rerio* alleles by PCR from fin clips, followed by Sanger sequencing to identify carriers or WT(ABb) or *edn3b*^*vp*.*r30c1*^ alleles. For *hsp70l*-inducible Edn3b transgenes, transgenic siblings and non-transgenic controls were reared from stages DR through J under conditions of repeated daily heat shock (38°C, 1 h) [16,23].

### CRISPR/Cas9 mutagenesis, transgenesis and clonal analyses

For CRISPR/Cas9 mutagenesis, 1-cell stage embryos were injected with T7 guide RNAs and Cas9 protein (PNA Bio) using standard procedures [74]. Guides were tested for mutagenicity by Sanger sequencing and injected fish were reared through adult stages at which time they were intercrossed to generate heteroallelic F1s from which single allele strains were recovered. CRISPR gRNA targets (excluding proto-spacer adjacent motif) were: *edn3a*^*b1282*^, GCCAGCTCCTGAAACCCCAC; *edn3b*^*vp*.*r30c1*^, GAGGATAAATGTACTCACTG; *edn3b*^*b1283*^, GGATAAATGTACTCACTGTG.

For transgenesis, constructs were generated using the Tol2Kit and Gateway cloning [75] and injected by standard methods with *Tol2* transposase mRNA [76]. For Edn3b-containing transgenes, F0 mosaic adults were screened for germline transmission and progeny tested for *hsp70l*-induction of linked fluorophore. Clonal analyses used mosaic F0 larvae and limiting amounts of *pnp4a*:*palmEGFP* transgene to insure that integrations were rare between and within individuals so that only single clones were likely to be labeled [35,36]. Sparsity of transgene+ embryos and similarity of starting clone sizes within such embryos between species suggests that labeling was indeed clonal. Transgene+ individuals were imaged at stages PR+ and J++.

### Sequences, genotyping, RT-PCR, and quantitative RT-PCR

Accession numbers for *D*. *rerio* and *D*. *nigrofasciatus edn3b* are NM_01311213 and MH705096. For distinguishing *D*. *rerio* wild-type and mutant alleles in hybrids we sequenced across induced lesions using primers edn3b*: F-TGCACTCATCTCCAGTCTTCTC, R-GTGTGACAGCGAAAGAGTAACG. For assessing persistence of transcript in wild-type and mutant backgrounds of *D*. *rerio*, we amplified *edn3b* and control cDNAs using primer sets: edn3b.Dr-c238, F-TTGGACATCAGCAGAAAGAAGC, R-CATAAGCAGCGACGAAGAACC; actb2: F-ACTGGGATGACATGGAGAAGAT, R-GTGTTGAAGGTCTCGAACATGA.

For assessing *edn3b* transcript abundance quantitatively across species, skins were harvested from stage-matched *D*. *rerio*, *D*. *nigrofasciatus* and *D*. *tinwini* and total RNAs isolated by Trizol (ThermoFisher) extraction as previously described [23]. For RT-PCR, first strand cDNAs were synthesized with SuperScript III reverse transcriptase (ThermoFisher) and oligo-dT primed First strand cDNAs were synthesized with iScript and oligo-dT priming (BioRad) and analyzed on an ABI StepOne Plus real time PCR instrument using custom designed Taqman probes against target sequence shared by *D*. *rerio* and *D*. *nigrofasciatus* (identical to *D*. *tinwini*). *edn3b* expression was normalized to that of *rpl13a*; normalization to a conserved *actb1* amplicon (ThermoFisher assay ID #Dr03432610_m1) yielded equivalent results in pilot analyses. Expression levels were assessed using the 2^–ΔΔCt^ method [77] with *D*. *rerio* expression levels set to 1. Comparisons of species differences in expression were repeated 4 times (with 2–4 biological replicates each) using matched stages of fish between DR+ and J. We did not detect significant differences between replicates/stages, or species x replicate/stage interactions, and so present normalized values across all replicates in the text. For analyzing allele-specific expression in hybrids, custom Taqman probes were designed to amplify an *edn3b* target from both species alleles, or from only *D*. *rerio* (*Dr*) or *D*. *nigrofasciatus* (*Dn*). Amplifications of *Dr* and *Dn* probes were normalized to that of the *Dr*, *Dn* probe. Hybrid samples included a total of 4 biological replicates. Primers (F, R) and target probes (T) were: *edn3b* (AIWR3Z6): F-CAGAGAATGTGTTTATTACTGTCATTTGGG, R-CCAAGGTGAACGTCCTCTCA, P-FAM-CTGGATCAACACCCCACAACG; *edn3b* (AI20TXP, *Dr*): F-TGGTGGTTCCAGCAGTGTTG, R-TGTGAGCGTGTGATGCTGAA, P-FAM-CAAGCTTCGCTTCTTTC; *edn3b* (AI1RVRH, *Dn*): F-GCTCTTTTGCTAATTGTGAGTTTGGT, R-ACCAGAGAAGACTGGAGATGAGT, P-FAM-CTCCTGCACTTGAAAAC; *rpl13a* (*Dr*, *Dn*): F-CAGAGAATGTGTTTATTACTGTCATTTGGG, R-CCAAGGTGAACGTCCTCTCA, P-FAM-CTGGGATCAACACCCCACAACG. Underlined bases are specific to the targeted species.

### Imaging

Images were acquired on: Zeiss AxioObserver inverted microscopes equipped either with Axiocam HR or Axiocam 506 color cameras or a Yokogawa laser spinning disk with Evolve camera, and an AxioZoom v16 stereomicroscope with Axiocam 506 color camera, all running ZEN blue software. An Olympus SZX12 stereomicroscope with Axiocam HRc camera and Axiovision software was additionally used for some imaging. Images were corrected for color balance and adjusted for display levels as necessary with all treatments or species within analyses treated identically. Images of swimming fish were captured with a Nikon D800 digital SLR equipped with Nikon AF-S VR Micro-Nikkor f2.8 IF/ED lens.

Counts of melanophores and coverage by iridophores used regions of interest defined dorsally and ventrally by the margins of the flank, anteriorly by the anterior insertion of the dorsal fin and posteriorly by the posterior insertion of the anal fin. Only hypodermal melanophores contributing to stripes were included in analyses; dorsal melanophores and melanophores on scales were not considered. All melanophore counts were performed on fish that had been treated with epinephrine, which contracts melanosomes towards cell centers and facilitates the identification of individual cells [16]. For assessing iridophore coverage, total areas covered by dense interstripe iridophores were estimated as these account for the majority of total hypodermal iridophores and areas covered by sparse iridophores within stripe regions could not be reliably estimated from brightfield images. Cell counts and area determinations were made using ImageJ. Time-lapse analyses of iridophore behaviors followed [21] and were performed for 15 h with 5 min frame intervals on *D*. *nigrofasciatus* as well as *D*. *rerio* siblings homozygous or heterozygous for *edn3b*^*vp*.*r30c1*^. All iridophores initially within regions of interest were counted. Proliferating iridophores were evident as single cells that rounded-up and then divided to generate adjacent daughter cells. For statistical analyses, we compared the number of proliferating iridophores to the number of non-proliferating iridophores, calculated as total starting number less the number of cells that underwent division. Individual genotypes of larvae used for time-lapse imaging were assessed by Sanger sequencing across the induced lesion.

### Statistical analysis

All statistical analyses were performed using JMP 14.0.0 statistical analysis software (SAS Institute, Cary NC) for Apple Macintosh. For linear models, residuals were examined for normality and homoscedasticity and variables transformed as necessary to meet model assumptions [78].

## Supporting information

S1 Fig Expansion of iridophore clones differs between *D*. *rerio* and *D*. *nigrofasciatus*. Representative images for individuals of each species mosaic for iridophore reporter *pnp4a*:*palmEGFP* at an early stage of pattern formation, and a late stage, once patterns were complete. Dashed yellow lines indicate approximate regions of correspondence between early and late images and I1–I3 indicate primary through tertiary interstripes, if present; 1D, 1V, 2V indicate positions of stripes, if present. In each species, iridophores were present within interstripes, where they were densely packed, and within stripe, where they were loosely arranged. Inset 1, clonal derived early iridophores in primary interstripe of *D*. *rerio*. Inset 2, In some individuals, autofluorescent xanthophores (x) were apparent but were distinguishable from iridophores by differences in shape. Inset 3, early iridophores of *D*. *nigrofasciatus*. Inset 4, Examples of spindle-shaped “type-L” iridophores [79] present at low abundance in each species.
(TIF)

S2 Fig Induced mutations in *D*. *rerio* Edn3 loci. Panels show genomic structures of Edn3 loci with locations encoding the mature peptides (green) as well as local nucleotide and amino acid sequences. Untranslated regions are shown in brown. For *edn3a*, the *b1282* allele has a 43 bp deletion that removes 13 of 20 amino acids comprising the active Edn3a peptide, with the addition of 4 novel amino acids (red). For *edn3b*, two alleles were generated with deletions of existing nucleotides and insertion of new nucleotides (red) covering the splice donor site downstream of exon 2 (boxed), resulting in the addition of novel amino acids and premature stop codons (*). Both *vp*.*r30c1* and *b1283* are likely to be loss-of-function mutations as their phenotypes were indistinguishable and also resembled independently derived *edn3b* alleles having similar lesions at the same target site [55]. Consistent with this inference, RT-PCR for *edn3b* transcript on skins of adult fish showed expression in wild-type (wt) but not *edn3b*^*b1283*^ or *edn3b*^*vp*.*r30c1*^ mutants; no-templ, no template control. Open reading frames are in upper case and intronic sequence in lower case.
(TIF)

S3 Fig Pigment pattern defects of *edn3b* mutants but not *edn3a* mutants resemble *D*. *nigrofasciatus*. (A) Details of ventral patterns illustrating deficiency in peritoneal iridophores (arrowhead) in *D*. *rerio edn3a* mutants but not *edn3b* mutants or *D*. *nigrofasciatus*. (B) Defects in areas covered by iridophores and numbers of melanophores in heterozygous and homozygous *edn3b* mutant *D*. *rerio* (*F*_2,48_ = 292.6, *F*_2,48_ = 69.8, respectively; both *P*<0.0001). Shown are least squares means±SE after controlling for variation in standard length (SL; both *P*<0.0001). Different letters above bars indicate means significantly different in Turkey-Kramer post hoc comparisons. Values above bars indicate samples sizes.
(TIF)

S4 Fig Reduced *edn3b* expression in *D*. *tinwini* compared to *D*. *rerio*. (A) Pigment pattern of *D*. *tinwini*. (B) Species differences in skin *edn3b* expression during adult pattern development (*F*_2,7_ = 48.2, *P*<0.0001). Shared letters indicate bars not significantly different in post hoc Turkey HSD comparisons of means (*P*>0.05). Numbers in bars indicate biological replicates
(TIF)

S1 File Supplementary Information File. **Data Matrices**. Numerical data used for quantitative analyses.
(XLSX)

## References

[1] Parichy DM (2015) Advancing biology through a deeper understanding of zebrafish ecology and evolution. Elife 4: e05635. 10.7554/eLife.05635 PMC4373672 25807087

[2] Endler JA (1988) Sexual Selection and Predation Risk in Guppies. Nature 332: 593–594.

[3] Rosenthal GG, Ryan MJ (2005) Assortative preferences for stripes in danios. Animal Behaviour 70: 1063–1066.

[4] Engeszer RE, Wang G, Ryan MJ, Parichy DM (2008) Sex-specific perceptual spaces for a vertebrate basal social aggregative behavior. Proc Natl Acad Sci U S A 105: 929–933. 10.1073/pnas.0708778105 18199839 PMC2242707

[5] Price AC, Weadick CJ, Shim J, Rodd FH (2008) Pigments, patterns, and fish behavior. Zebrafish 5: 297–307. 10.1089/zeb.2008.0551 19133828

[6] Engeszer RE, Patterson LB, Rao AA, Parichy DM (2007) Zebrafish in the wild: a review of natural history and new notes from the field. Zebrafish 4: 21–40. 10.1089/zeb.2006.9997 18041940

[7] Tang KL, Agnew MK, Hirt MV, Sado T, Schneider LM, et al (2010) Systematics of the subfamily Danioninae (Teleostei: Cypriniformes: Cyprinidae). Mol Phylogenet Evol 57: 189–214. 10.1016/j.ympev.2010.05.021 20553898

[8] Arunachalam M, Raja M, Vijayakumar C, Malaiammal P, Mayden RL (2013) Natural history of zebrafish (Danio rerio) in India. Zebrafish 10: 1–14. 10.1089/zeb.2012.0803 23590398

[9] McCluskey BM, Postlethwait JH (2015) Phylogeny of Zebrafish, a "Model Species," within Danio, a "Model Genus". Mol Biol Evol 32: 635–652. 10.1093/molbev/msu325 25415969 PMC4327152

[10] Dooley CM, Mongera A, Walderich B, Nusslein-Volhard C (2013) On the embryonic origin of adult melanophores: the role of ErbB and Kit signalling in establishing melanophore stem cells in zebrafish. Development 140: 1003–1013. 10.1242/dev.087007 23364329

[11] McMenamin SK, Bain EJ, McCann AE, Patterson LB, Eom DS, et al (2014) Thyroid hormone-dependent adult pigment cell lineage and pattern in zebrafish. Science 345: 1358–1361. 10.1126/science.1256251 25170046 PMC4211621

[12] Budi EH, Patterson LB, Parichy DM (2011) Post-embryonic nerve-associated precursors to adult pigment cells: genetic requirements and dynamics of morphogenesis and differentiation. PLoS Genet 7: e1002044 10.1371/journal.pgen.1002044 21625562 PMC3098192

[13] Singh AP, Schach U, Nusslein-Volhard C (2014) Proliferation, dispersal and patterned aggregation of iridophores in the skin prefigure striped colouration of zebrafish. Nat Cell Biol 16: 607–614. 10.1038/ncb2955 24776884

[14] Parichy DM, Elizondo MR, Mills MG, Gordon TN, Engeszer RE (2009) Normal table of postembryonic zebrafish development: staging by externally visible anatomy of the living fish. Developmental Dynamics 238: 2975–3015. 10.1002/dvdy.22113 19891001 PMC3030279

[15] Frohnhofer HG, Krauss J, Maischein HM, Nusslein-Volhard C (2013) Iridophores and their interactions with other chromatophores are required for stripe formation in zebrafish. Development 140: 2997–3007. 10.1242/dev.096719 23821036 PMC3912879

[16] Patterson LB, Parichy DM (2013) Interactions with iridophores and the tissue environment required for patterning melanophores and xanthophores during zebrafish adult pigment stripe formation. PLoS Genet 9: e1003561 10.1371/journal.pgen.1003561 23737760 PMC3667786

[17] Parichy DM, Turner JM (2003) Temporal and cellular requirements for Fms signaling during zebrafish adult pigment pattern development. Development 130: 817–833. 12538511 10.1242/dev.00307

[18] Nakamasu A, Takahashi G, Kanbe A, Kondo S (2009) Interactions between zebrafish pigment cells responsible for the generation of Turing patterns. Proc Natl Acad Sci U S A 106: 8429–8434. 10.1073/pnas.0808622106 19433782 PMC2689028

[19] Hamada H, Watanabe M, Lau HE, Nishida T, Hasegawa T, et al (2014) Involvement of Delta/Notch signaling in zebrafish adult pigment stripe patterning. Development 141: 318–324. 10.1242/dev.099804 24306107 PMC3879813

[20] Mahalwar P, Walderich B, Singh AP, Nusslein-Volhard C (2014) Local reorganization of xanthophores fine-tunes and colors the striped pattern of zebrafish. Science 345: 1362–1364. 10.1126/science.1254837 25214630

[21] Eom DS, Bain EJ, Patterson LB, Grout ME, Parichy DM (2015) Long-distance communication by specialized cellular projections during pigment pattern development and evolution. Elife 4: e12401 10.7554/eLife.12401 26701906 PMC4764569

[22] Eom DS, Parichy DM (2017) A macrophage relay for long-distance signaling during postembryonic tissue remodeling. Science 355: 1317–1320. 10.1126/science.aal2745 28209639 PMC5836293

[23] Patterson LB, Bain EJ, Parichy DM (2014) Pigment cell interactions and differential xanthophore recruitment underlying zebrafish stripe reiteration and Danio pattern evolution. Nat Commun 5: 5299 10.1038/ncomms6299 25374113 PMC4224114

[24] McClure M (1999) Development and evolution of melanophore patterns in fishes of the genus Danio (Teleostei: Cyprinidae). J Morphol 241: 83–105. 10.1002/(SICI)1097-4687(199907)241:1<83::AID-JMOR5>3.0.CO;2-H 10398325

[25] Quigley IK, Manuel JL, Roberts RA, Nuckels RJ, Herrington ER, et al (2005) Evolutionary diversification of pigment pattern in Danio fishes: differential fms dependence and stripe loss in D. albolineatus. Development 132: 89–104. 10.1242/dev.01547 15563521

[26] Quigley IK, Turner JM, Nuckels RJ, Manuel JL, Budi EH, et al (2004) Pigment pattern evolution by differential deployment of neural crest and post-embryonic melanophore lineages in Danio fishes. Development 131: 6053–6069. 10.1242/dev.01526 15537688

[27] Quigley IK, Parichy DM (2002) Pigment pattern formation in zebrafish: a model for developmental genetics and the evolution of form. Microsc Res Tech 58: 442–455. 10.1002/jemt.10162 12242701

[28] Braasch I, Brunet F, Volff JN, Schartl M (2009) Pigmentation pathway evolution after whole-genome duplication in fish. Genome Biol Evol 1: 479–493. 10.1093/gbe/evp050 20333216 PMC2839281

[29] Braasch I, Schartl M (2014) Evolution of endothelin receptors in vertebrates. Gen Comp Endocrinol 209: 21–34. 10.1016/j.ygcen.2014.06.028 25010382

[30] Kelsh RN, Harris ML, Colanesi S, Erickson CA (2009) Stripes and belly-spots-A review of pigment cell morphogenesis in vertebrates. Semin Cell Dev Biol 20: 90–104. 10.1016/j.semcdb.2008.10.001 18977309 PMC2744437

[31] Saldana-Caboverde A, Kos L (2010) Roles of endothelin signaling in melanocyte development and melanoma. Pigment Cell Melanoma Res 23: 160–170. 10.1111/j.1755-148X.2010.00678.x 20128875 PMC2911366

[32] Hirobe T (2011) How are proliferation and differentiation of melanocytes regulated? Pigment Cell Melanoma Res 24: 462–478. 10.1111/j.1755-148X.2011.00845.x 21375698

[33] Mort RL, Jackson IJ, Patton EE (2015) The melanocyte lineage in development and disease. Development 142: 620–632. 10.1242/dev.106567 25670789 PMC4325379

[34] Parichy DM, Mellgren EM, Rawls JF, Lopes SS, Kelsh RN, et al (2000) Mutational analysis of endothelin receptor b1 (rose) during neural crest and pigment pattern development in the zebrafish Danio rerio. Dev Biol 227: 294–306. 10.1006/dbio.2000.9899 11071756

[35] Tu S, Johnson SL (2010) Clonal analyses reveal roles of organ founding stem cells, melanocyte stem cells and melanoblasts in establishment, growth and regeneration of the adult zebrafish fin. Development 137: 3931–3939. 10.1242/dev.057075 20980402 PMC2976278

[36] Tryon RC, Johnson SL (2012) Clonal and lineage analysis of melanocyte stem cells and their progeny in the zebrafish. Methods Mol Biol 916: 181–195. 10.1007/978-1-61779-980-8_14 22914941 PMC3630497

[37] Stebbins GL, Basile DV (1986) Phyletic Phenocopies: A Useful Technique for Probing the Genetic and Developmental Basis of Evolutionary Change. Evolution 40: 422–425. 10.1111/j.1558-5646.1986.tb00483.x 28556037

[38] Parichy DM, Johnson SL (2001) Zebrafish hybrids suggest genetic mechanisms for pigment pattern diversification in*Danio*. Dev Genes Evol 211: 319–328. 11466528 10.1007/s004270100155

[39] Hoekstra HE, Hirschmann RJ, Bundey RA, Insel PA, Crossland JP (2006) A single amino acid mutation contributes to adaptive beach mouse color pattern. Science 313: 101–104. 10.1126/science.1126121 16825572

[40] Rohner N, Bercsenyi M, Orban L, Kolanczyk ME, Linke D, et al (2009) Duplication of fgfr1 permits Fgf signaling to serve as a target for selection during domestication. Curr Biol 19: 1642–1647. 10.1016/j.cub.2009.07.065 19733072

[41] Harris MP (2012) Comparative genetics of postembryonic development as a means to understand evolutionary change. Journal of Applied Ichthyology 28: 306–315.

[42] Stern DL (2014) Identification of loci that cause phenotypic variation in diverse species with the reciprocal hemizygosity test. Trends Genet 30: 547–554. 10.1016/j.tig.2014.09.006 25278102

[43] Young JJ, Tabin CJ (2017) Saunders's framework for understanding limb development as a platform for investigating limb evolution. Dev Biol 429: 401–408. 10.1016/j.ydbio.2016.11.005 27840200 PMC5426996

[44] Leal F, Cohn MJ (2018) Developmental, genetic, and genomic insights into the evolutionary loss of limbs in snakes. Genesis 56. 10.1002/dvg.23077 29095557

[45] Johnson SL, Africa D, Walker C, Weston JA (1995) Genetic control of adult pigment stripe development in zebrafish. Dev Biol 167: 27–33. 10.1006/dbio.1995.1004 7851648

[46] Mayer TC, Maltby EL (1964) An experimental analysis of pattern development in lethal spotting and belted mouse embryos. Dev Biol 9: 269–286. 14138974 10.1016/0012-1606(64)90025-9

[47] Baynash AG, Hosoda K, Giaid A, Richardson JA, Emoto N, et al (1994) Interaction of endothelin-3 with endothelin-B receptor is essential for development of epidermal melanocytes and enteric neurons. Cell 79: 1277–1285. 8001160 10.1016/0092-8674(94)90018-3

[48] Woodcock MR, Vaughn-Wolfe J, Elias A, Kump DK, Kendall KD, et al (2017) Identification of Mutant Genes and Introgressed Tiger Salamander DNA in the Laboratory Axolotl, Ambystoma mexicanum. Sci Rep 7: 6 10.1038/s41598-017-00059-1 28127056 PMC5428337

[49] Long AD, Mullaney SL, Mackay TF, Langley CH (1996) Genetic interactions between naturally occurring alleles at quantitative trait loci and mutant alleles at candidate loci affecting bristle number in Drosophila melanogaster. Genetics 144: 1497–1510. 8978039 10.1093/genetics/144.4.1497 PMC1207703

[50] Lopes SS, Yang X, Muller J, Carney TJ, McAdow AR, et al (2008) Leukocyte tyrosine kinase functions in pigment cell development. PLoS Genet 4: e1000026 10.1371/journal.pgen.1000026 18369445 PMC2265441

[51] Mo ES, Cheng Q, Reshetnyak AV, Schlessinger J, Nicoli S (2017) Alk and Ltk ligands are essential for iridophore development in zebrafish mediated by the receptor tyrosine kinase Ltk. Proc Natl Acad Sci U S A 114: 12027–12032. 10.1073/pnas.1710254114 29078341 PMC5692561

[52] Opdecamp K, Kos L, Arnheiter H, Pavan WJ (1998) Endothelin signalling in the development of neural crest-derived melanocytes. Biochem Cell Biol 76: 1093–1099. 10392719

[53] Dupin E, Glavieux C, Vaigot P, Le Douarin NM (2000) Endothelin 3 induces the reversion of melanocytes to glia through a neural crest-derived glial-melanocytic progenitor. Proc Natl Acad Sci U S A 97: 7882–7887. 10884419 10.1073/pnas.97.14.7882 PMC16639

[54] Hirobe T (2001) Endothelins are involved in regulating the proliferation and differentiation of mouse epidermal melanocytes in serum-free primary culture. J Investig Dermatol Symp Proc 6: 25–31. 10.1046/j.0022-202x.2001.00001.x 11764281

[55] Kim IS, Heilmann S, Kansler ER, Zhang Y, Zimmer M, et al (2017) Microenvironment-derived factors driving metastatic plasticity in melanoma. Nat Commun 8: 14343 10.1038/ncomms14343 28181494 PMC5309794

[56] McKinney ML, McNamara KJ (1991) Heterochrony: the Evolution of Ontogeny New York, New York: Plenum Press.

[57] Miller CT, Beleza S, Pollen AA, Schluter D, Kittles RA, et al (2007) cis-Regulatory changes in Kit ligand expression and parallel evolution of pigmentation in sticklebacks and humans. Cell 131: 1179–1189. 10.1016/j.cell.2007.10.055 18083106 PMC2900316

[58] Linnen CR, Poh YP, Peterson BK, Barrett RD, Larson JG, et al (2013) Adaptive evolution of multiple traits through multiple mutations at a single gene. Science 339: 1312–1316. 10.1126/science.1233213 23493712 PMC3836219

[59] Hosoda K, Hammer RE, Richardson JA, Baynash AG, Cheung JC, et al (1994) Targeted and natural (piebald-lethal) mutations of endothelin-B receptor gene produce megacolon associated with spotted coat color in mice. Cell 79: 1267–1276. 8001159 10.1016/0092-8674(94)90017-5

[60] Kaelin CB, Xu X, Hong LZ, David VA, McGowan KA, et al (2012) Specifying and sustaining pigmentation patterns in domestic and wild cats. Science 337: 1536–1541. 10.1126/science.1220893 22997338 PMC3709578

[61] Li L, Li D, Liu L, Li S, Feng Y, et al (2015) Endothelin Receptor B2 (EDNRB2) Gene Is Associated with Spot Plumage Pattern in Domestic Ducks (Anas platyrhynchos). PLoS One 10: e0125883 10.1371/journal.pone.0125883 25955279 PMC4425580

[62] Dorshorst B, Molin AM, Rubin CJ, Johansson AM, Stromstedt L, et al (2011) A complex genomic rearrangement involving the endothelin 3 locus causes dermal hyperpigmentation in the chicken. PLoS Genet 7: e1002412 10.1371/journal.pgen.1002412 22216010 PMC3245302

[63] Shinomiya A, Kayashima Y, Kinoshita K, Mizutani M, Namikawa T, et al (2012) Gene duplication of endothelin 3 is closely correlated with the hyperpigmentation of the internal organs (Fibromelanosis) in silky chickens. Genetics 190: 627–638. 10.1534/genetics.111.136705 22135351 PMC3276631

[64] Kinoshita K, Akiyama T, Mizutani M, Shinomiya A, Ishikawa A, et al (2014) Endothelin receptor B2 (EDNRB2) is responsible for the tyrosinase-independent recessive white (mo(w)) and mottled (mo) plumage phenotypes in the chicken. PLoS One 9: e86361 10.1371/journal.pone.0086361 24466053 PMC3900529

[65] Dushane GP (1934) The origin of pigment cells in Amphibia. Science 80: 620–621. 10.1126/science.80.2087.620-a 17809156

[66] Dalton HC (1949) Developmental Analysis of Genetic Differences in Pigmentation in the Axolotl. Proceedings of the National Academy of Sciences 35: 277–283. 10.1073/pnas.35.6.277 PMC1063021 16588892

[67] Amores A, Force A, Yan YL, Joly L, Amemiya C, et al (1998) Zebrafish hox clusters and vertebrate genome evolution. Science 282: 1711–1714. 9831563 10.1126/science.282.5394.1711

[68] Dehal P, Boore JL (2005) Two rounds of whole genome duplication in the ancestral vertebrate. PLoS Biol 3: e314 10.1371/journal.pbio.0030314 16128622 PMC1197285

[69] Braasch I, Gehrke AR, Smith JJ, Kawasaki K, Manousaki T, et al (2016) The spotted gar genome illuminates vertebrate evolution and facilitates human-teleost comparisons. Nat Genet 48: 427–437. 10.1038/ng.3526 26950095 PMC4817229

[70] Braasch I, Schartl M, Volff JN (2007) Evolution of pigment synthesis pathways by gene and genome duplication in fish. Bmc Evolutionary Biology 7. 10.1186/1471-2148-7-74 PMC1890551 17498288

[71] Braasch I, Volff JN, Schartl M (2009) The endothelin system: evolution of vertebrate-specific ligand-receptor interactions by three rounds of genome duplication. Mol Biol Evol 26: 783–799. 10.1093/molbev/msp015 19174480

[72] Lorin T, Brunet FG, Laudet V, Volff JN (2018) Teleost Fish-Specific Preferential Retention of Pigmentation Gene-Containing Families After Whole Genome Duplications in Vertebrates. G3 (Bethesda) 8: 1795–1806. 29599177 10.1534/g3.118.200201 PMC5940169

[73] Braasch I, Peterson SM, Desvignes T, McCluskey BM, Batzel P, et al (2015) A new model army: Emerging fish models to study the genomics of vertebrate Evo-Devo. J Exp Zool B Mol Dev Evol 324: 316–341. 10.1002/jez.b.22589 25111899 PMC4324401

[74] Shah AN, Davey CF, Whitebirch AC, Miller AC, Moens CB (2015) Rapid reverse genetic screening using CRISPR in zebrafish. Nat Methods 12: 535–540. 10.1038/nmeth.3360 25867848 PMC4667794

[75] Kwan KM, Fujimoto E, Grabher C, Mangum BD, Hardy ME, et al (2007) The Tol2kit: A multisite gateway-based construction kit forTol2 transposon transgenesis constructs. Developmental Dynamics 236: 3088–3099. 10.1002/dvdy.21343 17937395

[76] Suster ML, Kikuta H, Urasaki A, Asakawa K, Kawakami K (2009) Transgenesis in zebrafish with the tol2 transposon system. Methods Mol Biol 561: 41–63. 10.1007/978-1-60327-019-9_3 19504063

[77] Livak KJ, Schmittgen TD (2001) Analysis of relative gene expression data using real-time quantitative PCR and the 2(-Delta Delta C(T)) Method. Methods 25: 402–408. 10.1006/meth.2001.1262 11846609

[78] Sokal RR, Rohlf FJ (1981) Biometry New York, New York: W. H. Freeman and Company.

[79] Hirata M, Nakamura K, Kanemaru T, Shibata Y, Kondo S (2003) Pigment cell organization in the hypodermis of zebrafish. Dev Dyn 227: 497–503. 10.1002/dvdy.10334 12889058

