# Supplementary figures and images for: Evolution of Endothelin signaling and diversification of adult pigment pattern in Danio fishes
Source: PLoS Genet. 2018 Sep 18;14(9):e1007538. doi: 10.1371/journal.pgen.1007538 (PMC6161917; doi:10.1371/journal.pgen.1007538)

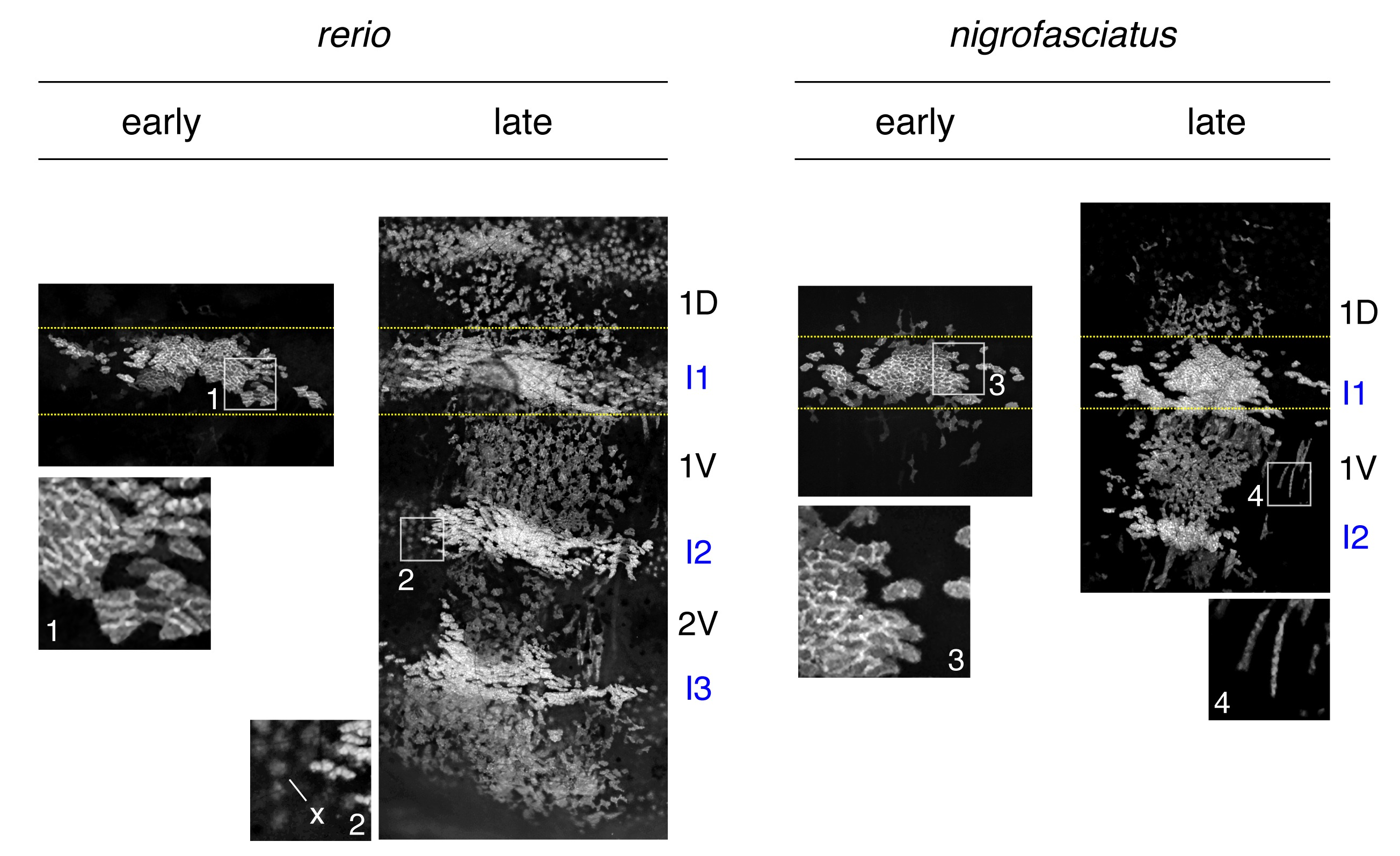

Supplement: S1 Fig — Representative images for individuals of each species mosaic for iridophore reporter pnp4a:palmEGFP at an early stage of pattern formation, and a late stage, once patterns were complete. Dashed yellow lines indicate approximate regions of correspondence between early and late images and I1–I3 indicate primary through tertiary interstripes, if present; 1D, 1V, 2V indicate positions of stripes, if present. In each species, iridophores were present within interstripes, where they were densely packed, and within stripe, where they were loosely arranged. Inset 1, clonal derived early iridophores in primary interstripe of D. rerio. Inset 2, In some individuals, autofluorescent xanthophores (x) were apparent but were distinguishable from iridophores by differences in shape. Inset 3, early iridophores of D. nigrofasciatus. Inset 4, Examples of spindle-shaped “type-L” iridophores [79] present at low abundance in each species. (TIF) [file pgen.1007538.s001.tif]

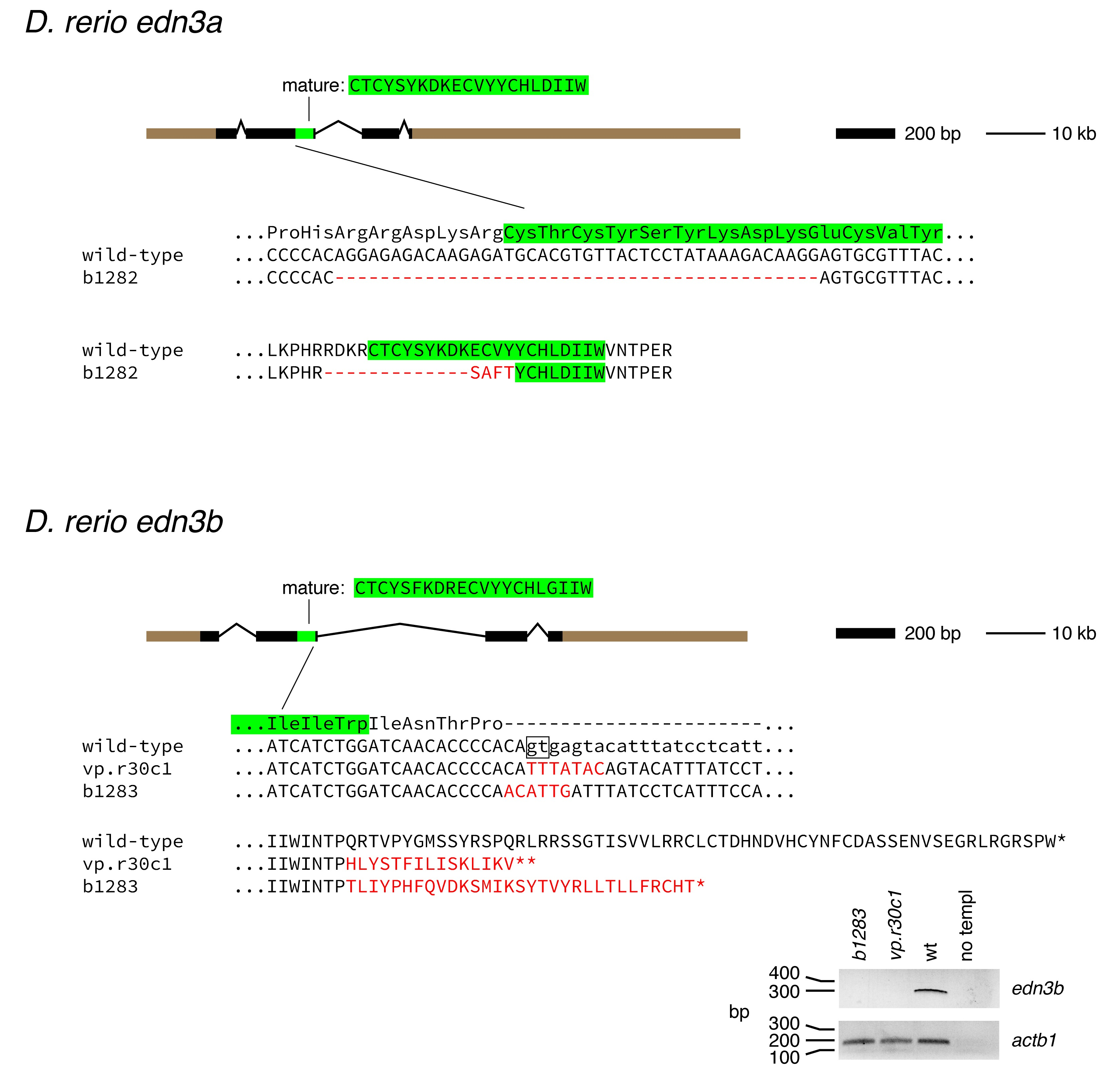

Supplement: S2 Fig — Panels show genomic structures of Edn3 loci with locations encoding the mature peptides (green) as well as local nucleotide and amino acid sequences. Untranslated regions are shown in brown. For edn3a, the b1282 allele has a 43 bp deletion that removes 13 of 20 amino acids comprising the active Edn3a peptide, with the addition of 4 novel amino acids (red). For edn3b, two alleles were generated with deletions of existing nucleotides and insertion of new nucleotides (red) covering the splice donor site downstream of exon 2 (boxed), resulting in the addition of novel amino acids and premature stop codons (*). Both vp.r30c1 and b1283 are likely to be loss-of-function mutations as their phenotypes were indistinguishable and also resembled independently derived edn3b alleles having similar lesions at the same target site [55]. Consistent with this inference, RT-PCR for edn3b transcript on skins of adult fish showed expression in wild-type (wt) but not edn3bb1283 or edn3bvp.r30c1 mutants; no-templ, no template control. Open reading frames are in upper case and intronic sequence in lower case. (TIF) [file pgen.1007538.s002.tif]

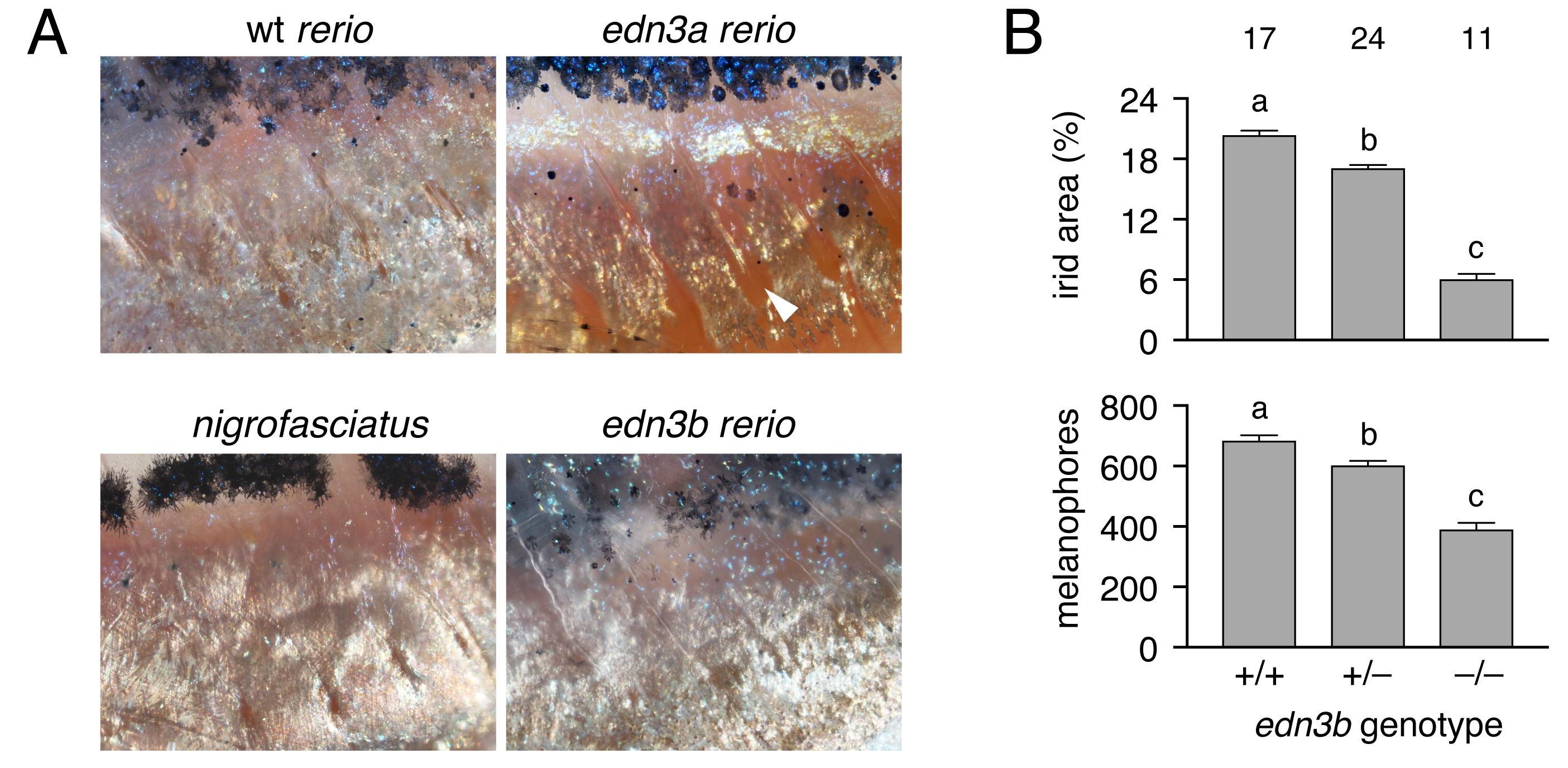

Supplement: S3 Fig — (A) Details of ventral patterns illustrating deficiency in peritoneal iridophores (arrowhead) in D. rerio edn3a mutants but not edn3b mutants or D. nigrofasciatus. (B) Defects in areas covered by iridophores and numbers of melanophores in heterozygous and homozygous edn3b mutant D. rerio (F2,48 = 292.6, F2,48 = 69.8, respectively; both P<0.0001). Shown are least squares means±SE after controlling for variation in standard length (SL; both P<0.0001). Different letters above bars indicate means significantly different in Turkey-Kramer post hoc comparisons. Values above bars indicate samples sizes. (TIF) [file pgen.1007538.s003.tif]

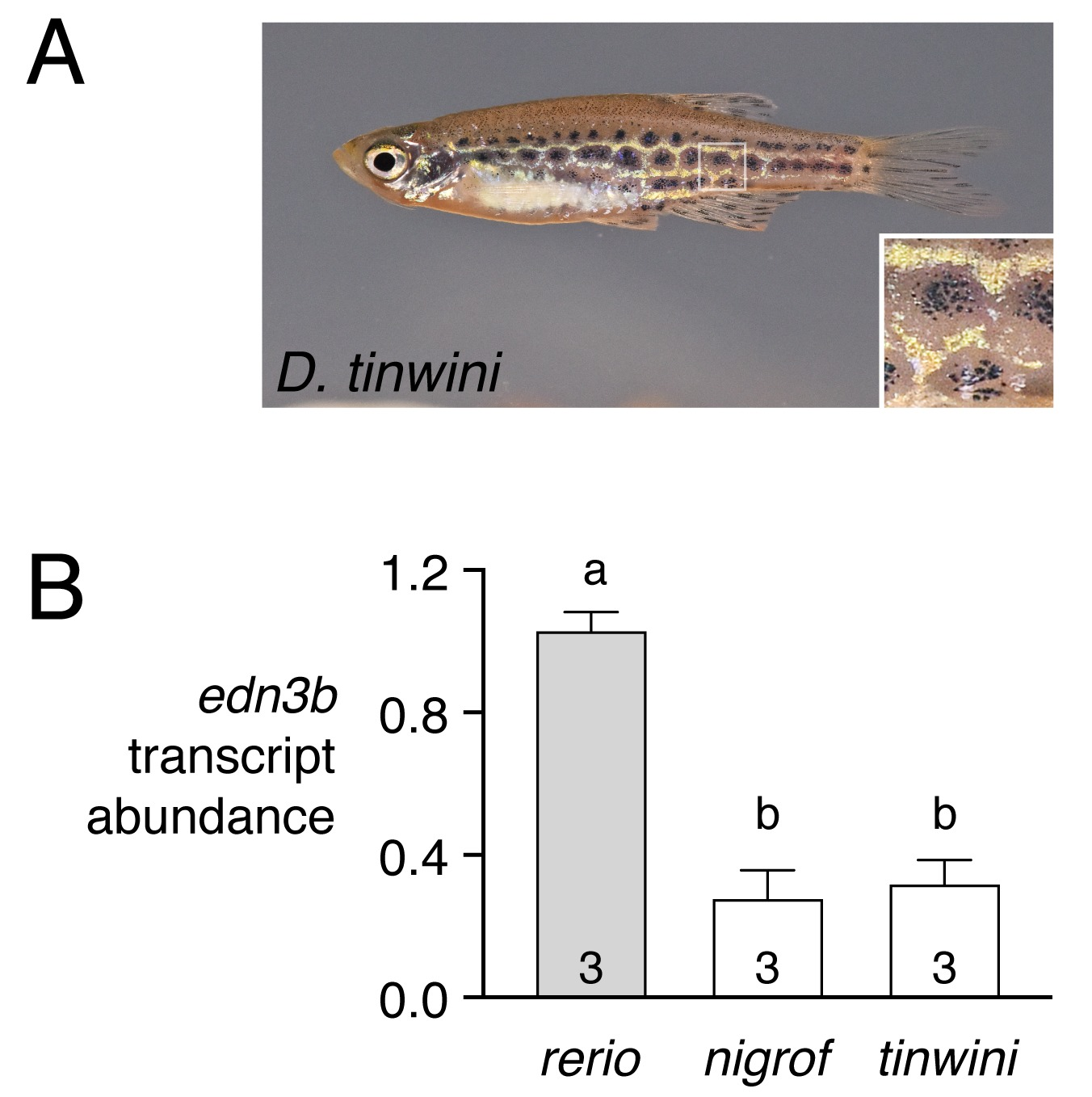

Supplement: S4 Fig — (A) Pigment pattern of D. tinwini. (B) Species differences in skin edn3b expression during adult pattern development (F2,7 = 48.2, P<0.0001). Shared letters indicate bars not significantly different in post hoc Turkey HSD comparisons of means (P>0.05). Numbers in bars indicate biological replicates (TIF) [file pgen.1007538.s004.tif]
